# Updating the Know Your Chances Website to Include Smoking Status as a Risk Factor for Mortality Estimates

**DOI:** 10.1001/jamanetworkopen.2023.17351

**Published:** 2023-06-08

**Authors:** Steven Woloshin, Victoria Landsman, Daniel G. Miller, Jeffrey Byrne, Barry I. Graubard, Eric J. Feuer

**Affiliations:** 1 Center for Medicine and the Media, The Dartmouth Institute for Health Policy and Clinical Practice, Geisel School of Medicine at Dartmouth, Lebanon, New Hampshire; 2 Lisa Schwartz Foundation for Truth in Medicine, Norwich, Vermont; 3 Institute of Work and Health and University of Toronto, Toronto, Ontario, Canada; 4 Information Management Services, Inc, Calverton, Maryland; 5 Division of Cancer Epidemiology and Genetics, National Cancer Institute, Rockville, Maryland; 6 Surveillance Research Program, Division of Cancer Control and Population Sciences, National Cancer Institute, Bethesda, Maryland

## Abstract

**Importance:**

To make wise decisions about the health risks they face, people need information about the magnitude of the threats as well as the context, such as how risks compare. Such information is often presented by age, sex, and race but rarely accounts for smoking status, a major risk factor for many causes of death.

**Objective:**

To update the National Cancer Institute’s Know Your Chances website to present mortality estimates for a broad set of causes of death and all causes combined by smoking status in addition to age, sex, and race.

**Design, Setting, and Participants:**

In this cohort study, mortality estimates using life table methods were calculated with the National Cancer Institute’s DevCan software package, combining data from the US National Vital Statistics System, National Health Interview Survey–Linked Mortality Files, National Institutes of Health–AARP (American Association of Retired Persons), Cancer Prevention Study II, Nurses’ Health and Health Professions follow-up studies, and Women’s Health Initiative. Data were collected from January 1, 2009, to December 31, 2018, and analyzed from August 27, 2019, to February 28, 2023.

**Main Outcomes and Measures:**

Age-conditional probabilities of dying due to various causes and all causes combined, accounting for competing causes of death, for people aged 20 to 75 years over the next 5, 10, or 20 years by sex, race, and smoking status.

**Results:**

A total of 954 029 individuals aged 55 years or older (55.8% women) were included in the analysis. Regardless of sex or race, for never-smokers, coronary heart disease represented the highest 10-year chance of death after about 50 years of age, which is higher than for any malignant neoplasm. Among current smokers, the 10-year chance of death due to lung cancer was almost as high as for coronary heart disease in each group. For Black and White female current smokers aged from the mid-40s onward, the 10-year probability of death due to lung cancer was substantially higher than for breast cancer. After 40 years of age, the observed effect of never vs current smoking on the 10-year chance of death due to all causes approximated adding 10 years of age. After 40 years of age when conditioning on smoking status, mortality risk for Black individuals was approximately that of White individuals 5 years older.

**Conclusions and Relevance:**

Using life table methods and accounting for competing risks, the revised Know Your Chances website presents age-conditional mortality estimates according to smoking status for a broad set of causes in the context of other conditions and all-cause mortality. The findings of this cohort study suggest that failing to account for smoking status results in inaccurate mortality estimates for many causes—namely, they are too low for smokers and too high for nonsmokers.

## Key Points

**Question:**

Do estimates of mortality due to various causes or all causes combined change when accounting for smoking status in addition to age, sex, and race?

**Findings:**

This cohort study used the National Cancer Institute’s Know Your Chances website to show that the chance of death due to major causes like heart disease, lung cancer, and all causes combined varies by smoking status. After 40 years of age, for example, the observed effect of never vs current smoking on the 10-year chance of death due to all causes approximates adding 10 years of age.

**Meaning:**

These findings suggest that mortality estimates failing to account for smoking status are too low for smokers and too high for nonsmokers.

## Teaser

This diagnostic study updates mortality risk charts from the National Cancer Institute to include current, former, and never smoking in relative risk for mortality.

## Introduction

To make wise decisions about the health risks they face, people need information about the magnitude of the threats as well as context to give the numbers meaning (eg, how risks compare). This information can inform patient-physician discussions and decisions about whether to try to reduce specific risks. Such information is also useful to policy makers, researchers, journalists and others attempting to understand, communicate, or respond to public health challenges.

Unfortunately, health risk messages often fail to adequately quantify risk magnitude or provide context. For example, for a segment during “Thyroid Cancer Awareness Month,” *Good Morning America*, one of the most-watched US morning television programs, stated that “more than 52 000 new cases will be reported this year.”^1^ This message format, presenting a large case count without the corresponding denominator, is a common strategy used to capture audience attention that may exaggerate the impression of risk; for example, 52 000 cases corresponds to a 0.2% chance (ie, 998 out of 1000 people will not get thyroid cancer this year). It also fails to provide a basic context that would help people decide how worried to be, such as the chance of dying from thyroid cancer or how thyroid cancer risk compares to other cancers or serious non–cancer-related health risks.

To make such information available, in 2015, the National Cancer Institute (NCI) launched Know Your Chances,^2^ a website allowing people to create tailored charts with the chance of death in user-specified time frames from a large set of cancer- and non–cancer-related deaths. As was typical, however, the charts presented mortality statistics according to age, sex, and race but did not account for smoking status. This matters because smoking is such a powerful risk factor for death. According to the Centers for Disease Control and Prevention (CDC), smoking remains the single largest cause of preventable disease and death in the US and causes 87% of deaths due to lung cancer, 32% of deaths due to coronary heart disease, and 79% of all cases of chronic obstructive pulmonary disease (COPD).^3^

Failing to account for smoking status means that the mortality risk for many causes of death (and all-cause mortality) is underestimated for smokers and overestimated for nonsmokers. Recognizing this, the NCI is in the process of updating their website using a new model to create charts for current, former, and never smokers. In this report, we present the methods we used to create the site and results generated. This work builds on the original proposal for risk charts by Woloshin et al^4^ but uses improved methods (eg, better accounting for competing risks), an expanded set of relative risks (RRs), and more recent data.

## Methods

### Overview

**Figure 1.**
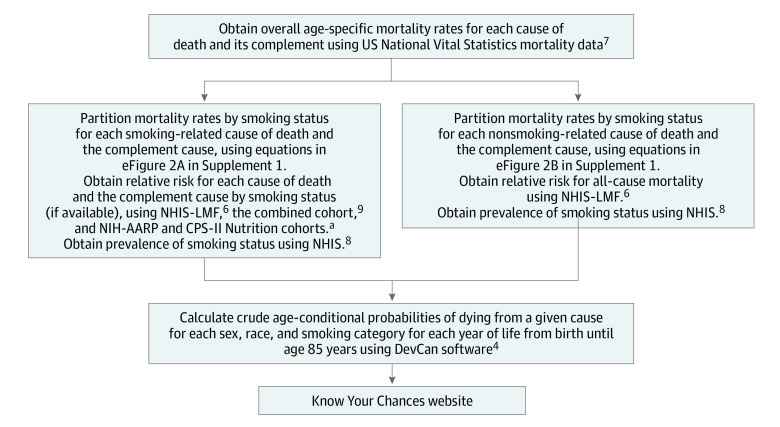
Overview of Data Sources and 4 Main Steps Used to Calculate Crude Age-Conditional Probabilities of Dying Due to a Given Cause and All Causes Combined by Race, Sex, Age, and Smoking Status Smoking status is categorized as current (smoked >100 cigarettes in their lifetime and smoked within past 2 years at the time of interview), former (smoked >100 cigarettes in their lifetime but quit >2 years before the interview), and never (did not smoke 100 cigarettes in their lifetime). CPS-II indicates Cancer Prevention Study II; NHIS-LMF, National Health Interview Survey–Linked Mortality Files; and NIH-AARP, National Institutes of Health–American Association of Retired Persons Diet and Health Study. ^a^Relative risks were provided by Christina Newton, MPH, American Cancer Association (email communication, August 25, 2016).

The revised Know Your Chances website provides age-conditional probabilities of dying due to a given cause (accounting for competing causes) separately by sex (men and women), race (Black, White, and all races), and smoking status (current, former, and never). These probabilities were calculated by the US NCI DevCan software, which computes estimates of diagnosis and death due to many specific causes from any age through 85 years.^5^ DevCan calculations are described in eMethods 1 in Supplement 1. To estimate risks of dying by smoking status, additional calculations are conducted before entry into DevCan by using multiple data sources,^6,7,8,9^ as detailed below and summarized in Figure 1. For this cohort study, we used all data that is in the public domain and summary statistics from published reports. The only exception is the use of the National Health Interview Survey–Linked Mortality Files (NHIS-LMF), where we submitted a study plan to gain access to their Research Data Center. The National Center for Health Statistics’ staff vet any estimates taken from the Research Data Center (which in our case were RRs and their SEs) to determine whether they can be publicly released (ie, not identifiable). Therefore, our project did not require institutional review board approval or informed consent. We followed the Strengthening the Reporting of Observational Studies in Epidemiology (STROBE) reporting guideline.

### Obtaining Overall Age-Specific Mortality Rates for Each Cause of Death and Its Complement

The 2 inputs needed to obtain overall age-specific mortality rates for each cause of death are (1) age-specific mortality rates for a particular cause of death and age interval and (2) complementary age-specific mortality rates (ie, all causes of death except the one under consideration). Inputs were obtained from the US National Vital Statistics System mortality data for years 2016 to 2018 containing counts of cause-specific deaths in 18 age catergories, (ie, <1, 1-4, 5-9, through 80-84 years) stratified by sex (male and female) and race (Black, White, and all races combined, typically obtained from a family member).^7,10^ All-cause mortality and the 87 causes of death used are found in eTable 1 in Supplement 1. This list includes 32 smoking-related^9,11^ and 55 major non–smoking-related causes from the National Center for Health Statistics’ list of 113 selected causes of death.^12^

### Partitioning Mortality Rates by Smoking Status for Smoking-Related Causes and the Complement Cause

For all-cause and 32 smoking-related causes of death, the mortality rates were partitioned by the 3 levels of smoking status by sex and race (details are provided in eFigure 2A in Supplement 1 and Landsman et al^13^). Briefly, for each cause of death, the overall age-specific mortality rate is the mean of the age-specific rates by smoking status weighted by the population prevalence of each smoking status:

Overall Mortality Rate = (Mortality Rate for Current Smokers × Prevalence for Current Smokers) + (Mortality Rate for Former Smokers × Prevalence for Former Smokers) + (Mortality Rate for Never Smokers × Prevalence for Never Smokers)

We solved the equation by substituting the RR for current or former vs never smokers and using an estimated prevalence of smoking status, for example:

Mortality Rate for Current Smokers = Mortality Rate for Never Smokers × RR for Death for Current vs Never Smokers

#### Obtaining Prevalence of Smoking Status

Age-specific smoking status prevalence was estimated from the NHIS,^8^ an annual, nationally representative, cross-sectional household survey of the US civilian noninstitutionalized population. We combined data for January 1, 2009, through December 31, 2018, restricted to individuals 40 years or older to achieve stable estimates across the age, race, and sex groups. The NHIS has less than 1% missing data on smoking status.

Smoking status was defined as follows. Current smokers smoked at least 100 cigarettes in their life and still smoked at the time of their interview^14^; former smokers smoked at least 100 cigarettes but quit. Because smoking relapse rates in the first 2 years may be greater than 60%,^15^ we reclassified smokers who quit less than 2 years before their interview as current smokers to prevent contamination of former smokers from recent (unstable) quitters. Never smokers smoked fewer than 100 cigarettes in their lifetime.

#### Obtaining RRs for Each Cause of Death and Its Complement by Smoking Status

The RRs for dying of smoking-related causes (eTables 2 and 3 in Supplement 1) were obtained from various sources to ensure the most reliable estimates. Where possible, we used the NHIS-LMF cohort because it is a nationally representative cohort of the US noninstitutionalized population with follow-up using the National Death Index to obtain cause of death.^6^ We used this cohort to estimate RRs for 10 of 32 smoking-related causes of death with at least 50 deaths observed for each smoking category (race was based on the interviewer’s observation). We used data for individuals 40 years or older surveyed in 1994 to 1995 (1996 NHIS did not include every variable needed in our models) and 1997 to 2013 with follow-up through 2015, with a total sample size of 529 804 individuals. Due to small numbers of deaths at younger than 40 years, we set the RR to 1 in this age range (eMethods 2 in Supplement 1).

Due to small numbers of deaths in the NHIS-LMF cohort, we used published pooled data from 5 cohorts of US volunteers (ie, combined cohort of 421 378 men and 532 651 women 55 years or older, approximately 90% White) to obtain RRs for the other 22 smoking-related causes (details are provided in eTable 2 in Supplement 1; race is based on self-report).^9^ Missing data about smoking in the combined cohort are low at approximately 3.8% for the National Institutes of Health–AARP (American Association of Retired Persons), 13% for the Cancer Prevention Study II, and 1.3% for the Women’s Health Initiative and not applicable for the Nurses Health and Health Profession cohorts.^16^

There was minimal loss to follow-up for mortality for any of these cohorts and the NHIS because they link to the National Death Index, a comprehensive database of all recorded deaths in the US. All estimates using NHIS-LMF data were survey weighted.

### Partitioning of Mortality Rates by Smoking Status for Non–Smoking-Related Causes and Their Complements

For non–smoking-related causes of death, we assumed that age-specific mortality rates for all smoking categories equaled the overall age-specific mortality rate. However, for each cause, mortality rates for the complementary causes are partitioned, using the same approach as above, because they include some smoking-related causes (eg, deaths due to accidents are not smoking-related, but deaths due to all causes other than accidents are since they include causes like lung cancer). Partition methods are summarized in eFigure 2B in Supplement 1.

### Calculation of Crude Age-Conditional Probabilities of Dying Due to a Given Cause for Each Sex, Race, and Smoking Category for Each Year of Life From Birth to 85 Years of Age

The 2 sets of age-specific rates for 18 age categories (<1, 1-4, 5-9, through 80-84 years) were calculated for (1) mortality rates for any given cause of death and (2) mortality rates for the corresponding complement causes of death. The calculations terminated at the interval ending at 84 years of age since causes of death are less accurate at older ages and, therefore, the estimated RRs necessary to partition the mortality are less reliable.

Data were analyzed from August 27, 2019, to February 28, 2023. All analyses used R core software, version 4.2.2 (R Foundation for Statistical Computing).

## Results

### Smoking Status and Mortality

A total of 954 029 individuals aged 55 years or older (55.8% women and 44.2% men) were included in the analysis. For smoking-related causes of death, mortality estimates that do not account for smoking status consistently underestimate risk for smokers and overestimate it for nonsmokers (and to a lesser extent, former smokers). For example, the chance that a White man aged 60 years will die in the next 10 years due to any cause is 14.5% (95% CI, 14.4%-14.5%) with all smoking categories combined, but the chance is 9.7% (95% CI, 9.4%-10.0%) for a never smoker, 13.2% (95% CI, 12.9%-13.5%) for a former smoker, and 27.3% (95% CI, 26.7%-27.9%) for a current smoker (Table 1). Similar patterns are seen across age, sex, and race.

**Table 1.** Chance of Death in the Next 10 Years for Black or White Men and Women Aged 60 Years Due to Selected Smoking- and Non–Smoking-Related Causes, Overall and by Smoking Status

Cause of death	Chance of death by smoking category, %			
	Combined^a^	Never	Former^b^	Current
**Men**				
Smoking-related causes				
All causes combined				
Black	21.3	13.6	18.2	36.5
White	14.5	9.7	13.2	27.3
Lung cancer				
Black	1.6	0.4	1.0	4.2
White	1.2	0.3	0.9	3.8
Coronary heart disease				
Black	4.2	2.8	3.8	6.8
White	2.8	2.0	2.7	5.0
Non–smoking-related causes				
Accidents				
Black	1.0	1.1	1.0	0.9
White	0.6	0.7	0.7	0.6
Septicemia				
Black	0.4	0.4	0.4	0.4
White	0.2	0.2	0.2	0.2
**Women**				
Smoking-related causes				
All causes combined				
Black	13.1	9.6	13.2	25.6
White	9.3	6.7	9.3	18.5
Lung cancer				
Black	0.8	0.2	0.8	2.9
White	0.9	0.2	1.0	3.4
Coronary heart disease				
Black	2.1	1.6	2.2	3.8
White	1.2	0.9	1.2	2.1
Non–smoking-related causes				
Accidents				
Black	0.3	0.3	0.3	0.3
White	0.3	0.3	0.3	0.3
Septicemia				
Black	0.3	0.3	0.3	0.3
White	0.2	0.2	0.2	0.2

^a^ Data for smoking categories combined are from the Know Your Chances website.^2^

^b^ Indicates smoked more than 100 cigarettes in their lifetime and quit at least 2 years before interview.

### Chance of Death by Age, Sex, Race, and Smoking Status

Table 2 shows the probabilities of death over the next 10 years of selected major causes for Black female never and current smokers in 5-year age categories beginning at 30 years of age (figures for former smokers generally fall between those for never and current smokers). Corresponding tables for other race and sex categories are found in eTables 4, 5, and 6 in Supplement 1; tables with all included causes and for those 20 years and older can be created on the Know Your Chances website.^2^

**Table 2.** Chance of Dying in the Next 10 Years by Smoking Status for Black Women Starting at Ages 30 to 70 Years^a^

Cause of death	Smoking status	Probability of death by risk interval, %								
		30 to <40 y	35 to <45 y	40 to <50 y	45 to <55 y	50 to <60 y	55 to <65 y	60 to <70 y	65 to <75 y	70 to <80 y
All causes^b^	Never	1.4	1.8	2.4	3.6	4.9	6.8	9.6	13.8	20.4
	Current	1.6	3.0	5.1	7.8	12.3	18.5	25.6	34.7	44.4
Vascular disease										
Abdominal aortic aneurysm	Never	<0.1	<0.1	<0.1	<0.1	<0.1	<0.1	<0.1	<0.1	<0.1
	Current	<0.1	<0.1	<0.1	<0.1	<0.1	<0.1	<0.1	0.1	0.1
Coronary heart disease	Never	0.1	0.1	0.2	0.4	0.6	1.0	1.6	2.5	3.9
	Current	0.1	0.3	0.7	1.1	1.9	3.0	3.8	4.8	6.2
Heart failure	Never	<0.1	<0.1	<0.1	0.1	0.1	0.2	0.3	0.4	0.7
	Current	<0.1	<0.1	0.1	0.1	0.2	0.3	0.4	0.7	1.0
High blood pressure^c^	Never	0.1	0.1	0.2	0.2	0.3	0.4	0.6	0.8	1.1
	Current	0.1	0.1	0.3	0.4	0.6	0.7	1.0	1.2	1.7
Stroke	Never	<0.1	0.1	0.1	0.2	0.3	0.4	0.7	1.1	1.8
	Current	<0.1	0.1	0.2	0.3	0.4	0.6	1.0	1.5	2.3
Cancer										
Breast	Never	0.1	0.1	0.2	0.3	0.4	0.5	0.6	0.7	0.8
	Current	0.1	0.2	0.3	0.4	0.5	0.6	0.8	0.9	0.9
Cervical	Never	<0.1	<0.1	<0.1	<0.1	0.1	0.1	0.1	0.1	0.1
	Current	<0.1	<0.1	<0.1	0.1	0.1	0.1	0.1	0.1	0.1
Colon or rectum	Never	<0.1	<0.1	0.1	0.1	0.2	0.3	0.3	0.4	0.4
	Current	<0.1	<0.1	0.1	0.2	0.2	0.3	0.4	0.5	0.6
Lung and bronchus	Never	<0.1	<0.1	<0.1	0.1	0.1	0.2	0.2	0.3	0.4
	Current	<0.1	0.1	0.2	0.5	1.2	2.1	3.2	4.7	5.8
Ovarian	Never	<0.1	<0.1	<0.1	0.1	0.1	0.1	0.2	0.2	0.3
	Current	<0.1	<0.1	<0.1	<0.1	0.1	0.1	0.2	0.2	0.2
Pancreatic	Never	<0.1	<0.1	<0.1	0.1	0.1	0.2	0.3	0.4	0.5
	Current	<0.1	<0.1	0.1	0.1	0.2	0.4	0.5	0.7	0.9%
COPD	Never	<0.1	<0.1	<0.1	<0.1	<0.1	0.1	0.1	0.2	0.3
	Current	<0.1	<0.1	0.1	0.3	0.6	1.1	1.7	2.7	3.9
Infections										
AIDS	Never	<0.1	0.1	0.1	0.1	0.1	0.1	0.1	0.1	<0.1
	Current	<0.1	0.1	0.1	0.1	0.1	0.1	0.1	<0.1	<0.1
Pneumonia and/or flu^c^	Never	<0.1	<0.1	<0.1	0.1	0.1	0.1	0.2	0.3	0.4
	Current	<0.1	<0.1	0.1	0.1	0.2	0.2	0.3	0.5	0.7
Accidents and injury										
Accidents	Never	0.2	0.3	0.3	0.3	0.4	0.3	0.3	0.3	0.4
	Current	0.2	0.3	0.3	0.3	0.3	0.3	0.3	0.3	0.3
Homicide	Never	0.1	0.1	0.1	<0.1	<0.1	<0.1	<0.1	<0.1	<0.1
	Current	0.1	0.1	0.1	<0.1	<0.1	<0.1	<0.1	<0.1	<0.1
Suicide	Never	<0.1	<0.1	<0.1	<0.1	<0.1	<0.1	<0.1	<0.1	<0.1
	Current	<0.1	<0.1	<0.1	<0.1	<0.1	<0.1	<0.1	<0.1	<0.1
Diabetes^c^	Never	0.1	0.1	0.1	0.2	0.3	0.5	0.7	1.0	1.3
	Current	0.1	0.1	0.2	0.2	0.4	0.5	0.7	1.0	1.3
Neurological diseases										
Alzheimer disease	Never	<0.1	<0.1	<0.1	<0.1	<0.1	<0.1	0.1	0.3	0.8
	Current	<0.1	<0.1	<0.1	<0.1	<0.1	<0.1	0.1	0.3	0.7
Parkinson disease	Never	<0.1	<0.1	<0.1	<0.1	<0.1	<0.1	<0.1	0.1	0.2
	Current	<0.1	<0.1	<0.1	<0.1	<0.1	<0.1	<0.1	<0.1	0.1

Abbreviation: COPD, chronic obstructive pulmonary disease.

^a^ Similar tailored charts can be created interactively on the Know Your Chances website^2^ for black men, white men and women, and men and women of all races combined. Data sources include the National Health Interview Survey (NHIS)–Linked Mortality Files,^6^ National Center for Health Statistics^8^ (using years 2016-2018) for mortality; NHIS for smoking prevalence; and combined cohort (relative risks).^9^ Risk estimates are based on the year range 2016 to 2018.

^b^ Percentages in columns do not total all-cause percentages because there are many other causes of death besides those listed.

^c^ Rule used by the National Center for Disease Statistics may result in overcounting and undercounting of some underlying causes of death. Diabetes and high blood pressure, for example, are probably undercounted because they are often reported as contributing rather than underlying factors due to uncertainty in the chain of death. Flu deaths are probably undercounted since many pneumonia deaths, unrelated to flu, get attributed to it. Since disentangling these conditions is difficult, we present a combined pneumonia and/or flu category.

#### White Male Never Smokers

The chance of dying in the next 10 years is highest for accidents and suicide at approximately 0.8% and 0.3%, respectively, for ages 20 to 45 years. For ages 50 years and older, this chance is highest for coronary heart disease (ie, 0.9% at 50 years of age; 4.9% at 70 years of age). The chance of death over the next 10 years from other major causes (eg, COPD, pneumonia) do not approximate that of accidents until after 70 years of age. Among malignant neoplasms, the chance of dying over the next 10 years of colon, lung, pancreatic, and prostate cancers are similar across all ages and do not approximate that of accidents until 70 years or older.

#### White Male Current Smokers

The highest chances of dying in the next 10 years are due to accidents and suicide until ages 35 and 45 years, respectively, at which point the chance of death due to coronary heart disease becomes greatest. The chance of dying due to coronary heart disease in the next 10 years rises substantially between 45 and 70 years of age from 1.4% to 8.1%, exceeding all other causes by a factor greater than 5 except for lung cancer (death approximates that for coronary heart disease at 65 years or older) and COPD (which rises substantially after 55 years of age and is similar to that for coronary heart disease by 70 years of age). Among malignant neoplasms, the chance of dying over the next 10 years of colon, pancreatic, and prostate cancers all have a similar order of magnitude, generally about one-tenth as large as that of lung cancer death regardless of age.

#### Black Male Never Smokers

The 10-year chance of death patterns for this group parallels that of their White counterparts, with few exceptions. For example, the 10-year chance of death due to homicide numerically exceeds that for accidents (eg, 0.9% vs 0.5% at 20 years of age for Black male never smokers and 0.7% vs 0.1% for White male never smokers). At 30 years of age, the 10-year chance of dying due to homicide is about 7 times higher for Black men than for White men (0.7% vs 0.1%). Coronary heart disease exceeds homicide and accidents and is the highest cause of death in the next 10 years after 40 years of age for current smokers and 50 years of age for never smokers. Among malignant neoplasms, for never smokers, the 10-year chances of death due to colon, pancreatic, lung, and prostate cancers are similar until 65 years of age, after which the 10-year chance of prostate cancer death becomes highest; for current smokers, lung cancer exceeds all other cancer-related deaths by 35 years of age and is about 4 to 5 times greater than for prostate cancer.

#### Black and White Women

The chance of dying in the next 10 years due to all causes was lower than for men with the same smoking status at all ages. For Black women, the 10-year chance of dying due to accidents exceeded all other causes until it was surpassed by coronary heart disease at 40 years of age for current smokers and 45 years of age for never smokers. For White women, the corresponding ages were 45 and 55 years, respectively.

#### White Female Never Smokers

The 10-year chance of death due to breast cancer was slightly higher than for any other cancer after 35 years of age. The chance of death due to coronary heart disease was similar to that for breast cancer until after 50 years of age and then exceeded it. These patterns were similar for Black female never smokers.

#### White Female Current Smokers

The highest 10-year chance of death was due to lung cancer after 45 years of age, exceeding that for coronary heart disease. The chance of death due to COPD exceeded that due to lung cancer by 70 years of age. The chance of death due to coronary heart disease was consistently higher than that due to breast cancer after 40 years of age (eg, 1.6% vs 0.4% at 55 years of age, and 4.3% vs 0.8% at 70 years of age). The chance of death due to lung cancer exceeded that for breast cancer from 45 years and older, (eg, 5.1% vs 0.6% at 65 years of age).

#### Black Female Never Smokers

The 10-year chance of death due to breast cancer was slightly higher than for any other cancer from 30 years and older. The chance of death due to coronary heart disease was similar to that for breast cancer until after 45 years of age and then exceeded it (see Table 2).

#### Black Female Current Smokers

The highest 10-year chance of death due to coronary heart disease was at 35 years and older. The chance was substantially higher than that for breast cancer (eg, 3.0% vs 0.6% at 55 years of age and 6.2% vs 0.9% at 70 years of age). The 10-year chance of death due to lung cancer death exceeded that for breast cancer from 45 years and older (eg, 2.1% vs 0.6% at 55 years of age and 5.8% vs 0.9% at 70 years of age). The figures for COPD closely approximated those for lung cancer.

### Smoking and Chance of Dying

After 40 years of age, the observed effect of never vs current smoking on the 10-year chance of death due to all causes was about the same as adding 10 or more years of age. For example, the 10-year chance of death due to all causes for a White male smoker aged 55 years was 19.7%, which was greater than the corresponding chance for a nonsmoker at 65 years of age (14.3%) and almost as large as that for a nonsmoker at 70 years of age (22.7%).

**Figure 2.**
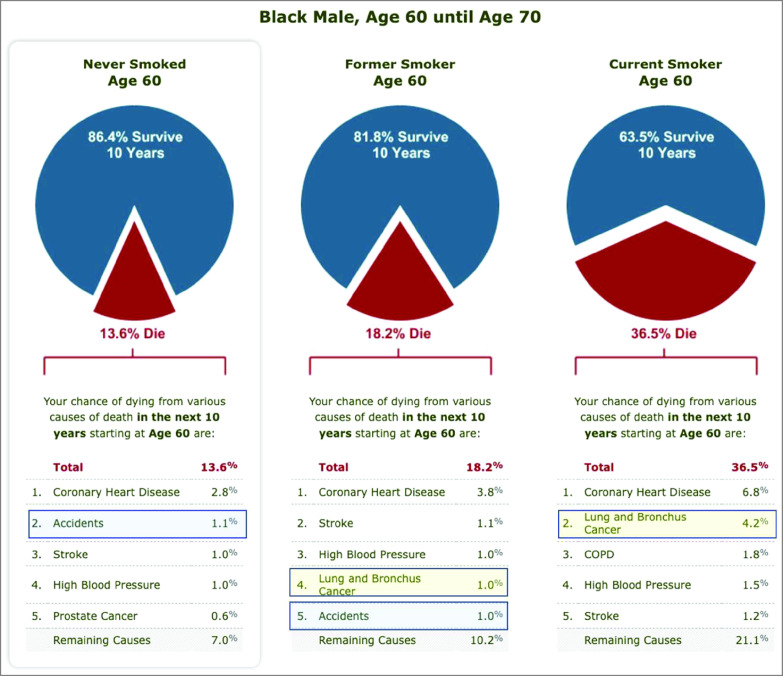
Causes of Death and All Causes Combined for Black Men Aged 60 to 70 Years From the Know Your Chances website.^2^

The effect of smoking status on the magnitude and order of causes of death is illustrated in Figure 2. For a Black male never smoker aged 60 years, lung cancer moved from below the tenth ranked cause to the fourth and second ranks as expected, given its strong association with smoking. Death due to accidents, which is not smoking related, moved in the opposite direction from second to fifth and seventh ranks as smoking-related causes displaced it. Of note, the 10-year chance of death due to accidents changed from 0.7% (for a never smoker) to 0.6% (for a current smoker). This does not mean that smoking protects against accidents; rather, in the presence of competing risks of death, as more current smokers die of smoking-related causes, fewer remain who could die of a non–smoking-related cause (effect similar for a corresponding White man [eFigure 1 in Supplement 1]). eMethods 3 in Supplement 1 contains a discussion of competing risks.

### Race and Chance of Dying

The magnitude of the 10-year chance of death due to all causes was substantially higher for Black (male or female) than for White individuals. After 40 years of age, the difference was approximately the same as adding 5 years of age to a corresponding White individual. For example, the 10-year chance of death due to all causes for a Black male never smoker aged 55 years was 9.6%, about the same as a White male never-smoker aged 60 years. For a Black female smoker aged 55 years, the chance was 18.5%, the same as for a White female smoker aged 60 years. The substantially increased 10-year chance of death due to all causes combined and coronary heart disease for Black vs White individuals is also highlighted in Table 1.

## Discussion

The updated Know Your Chances website makes it possible to create interactive risk charts with age-, sex-, and race-specific estimates of the probability of death due to a broad set of causes stratified by smoking status across variable time frames. The goal of the website is to help people make sense of the disease risks they face by providing basic facts and context: how big the various risks are and how they compare with each other. Estimates on the updated website represent a substantial improvement over typically available presentations that only provide mortality data for a few causes and fail to account for smoking status, meaning they are inaccurate for many causes and all causes of death combined, that is, too low for smokers and too high for nonsmokers, as shown in Table 1. For example, the CDC’s Health Effects of Smoking website notes that “smoking harms nearly every organ of the body, causes many diseases, and reduces the health of smokers in general.” However, the website quantifies risk for only a few conditions, and then only in relative terms, and does not stratify by age, race, or smoking status (ie, current vs former).^3^

Our charts highlight how patterns and probabilities of death change by smoking status and help illustrate why the CDC calls smoking “the single largest cause of preventable disease.”^3^ Consequently, the charts may be useful to inform efforts to limit smoking initiation or promote cessation. The charts also highlight important differences by race, but further research is needed to explain and address the underlying causes.

Creating the website entailed addressing a series of challenges. We relied on cause of death data from the National Vital Statistics System as a basic input to our calculations. While these are the best available US mortality data, they are derived from death certificates, and underlying cause of death attributions may not always be accurate, especially at older ages.^17^ Data availability also limited our ability to generate sufficiently precise estimates for races other than White and Black (nor could we account for ethnicity) or for individuals 85 years or older. We also required RRs for the various causes of death and all causes combined by smoking status. Historically, studies generating such RRs^9^ relied on large cohorts of volunteers to allow for precise estimates across population groups. These cohorts might limit generalizability because volunteers were more likely to be White and affluent compared with the general population. We therefore used NHIS-LMF data to calculate nationally representative RRs where possible for selected smoking-related causes. A sensitivity analysis (eMethods 4 in Supplement 1) comparing nationally representative with combined cohort–derived RRs^9^ was similar except for 2 statistically significant differences: the RR for death due to lung cancer death for men is 25.0 (combined cohort) vs 14.4 (NHIS-LMF cohort), and the RR for death due to COPD for women is 25.0 (combined cohort) vs 19.5 (NHIS-LMF cohort). The differences observed may be an indication of volunteer selection bias, suggesting the US nationally representative samples should be preferred where feasible.

Importantly, like the earlier versions, the updated website does not include CIs with risk estimates. This is similar to other heavily used and cited risk-modeling websites, such as the NCI Breast Cancer Risk Assessment Tool.^18^ Reasons for doing so include concerns about table readability and open questions about how to present CIs in a way that would not be potentially confusing for the general public. Because some users might want access to CIs to help them understand and use our estimates, we plan to provide them in a website supplement and are actively developing the complicated statistical methodology needed (ie, to combine the various data sources).

### Strengths and Limitations

Due to time lags in national data availability, the website figures predate the beginning of the COVID-19 pandemic. A website strength is that it will be updated annually (age-specific mortality rates) and periodically (smoking prevalence estimates and RRs). It is not clear what to do about unusual and (we hope) time-limited events like COVID-19. Incorporating a major new mortality risk into our charts only makes sense if the risk is fairly stable over time. Deciding what to do depends on the trajectory of the COVID-19 pandemic.

Finally, while the charts account for arguably the 3 most important risk factors for death—age, sex, and smoking—they do not account for others such as drug or alcohol use, obesity, family history, genetic risks, or presence of a variety of diseases. In theory, charts tailored to a wide variety of risk factors might provide more personalized risk estimates. However, reliable risk models are not available for many of the risk factors alone or in combinations, and, consequently, extensive tailoring would introduce substantially more uncertainty. Our estimates would serve as useful inputs to researchers attempting to create more personalized estimates.

## Conclusions

Using life table methods and accounting for competing risks, the revised Know Your Chances website presents age-conditional mortality estimates for a broad set of causes that will help the public, policy makers, journalists, researchers, and others appreciate the magnitude of various health risks they face and put the risks in the context of other conditions and all-cause mortality. The present prognostic study demonstrates that failing to account for smoking results in inaccurate estimates for many causes of death (ie, too low for smokers and too high for nonsmokers).

## Notes

Supplement 1. **eMethods 1.** Double Decrement Lifetable **eMethods 2.** Estimating the RRs of Dying by Smoking Status From the NHIS-LMF Cohort Using Cox Proportional Hazards Model **eMethods 3.** Counterintuitive Competing Risks Example **eMethods 4.** Comparison Between the RRs Obtained From NHIS-LMF Cohort vs Combined Cohort for Selected Causes of Death **eTable 1.** Causes of Death Used on Know Your Chances Website by Association With Smoking Status and *ICD-10* Codes **eTable 2.** Estimated Relative Risks (RRs) and Their Corresponding 95% CIs for All-Cause Mortality and 32 Smoking-Related Causes of Death **eTable 3.** Relative Risks (RRs) for Complement Causes of Death (ie, All-Cause Minus the Cause of Interest) for 10 Causes of Deaths From NHIS-LMF **eTable 4.** Chance of Dying in the Next 10 Years by Smoking Status for Black Men **eTable 5.** Chance of Dying in the Next 10 Years by Smoking Status for White Men **eTable 6.** Chance of Dying in the Next 10 Years by Smoking Status for White Women **eFigure 1.** The Effect of Smoking Status on the Order of the Top 5 Causes of Death and Absolute Risk of Death Over 10 Years for a 60-Year-Old White Man **eFigure 2.** Overview of Computational Details for Partitioning Mortality Rates by Smoking Status for Smoking-Related and Non–Smoking-Related Causes of Death
eReferences

Supplement 2.
Data Sharing Statement
