# Supplementary material for: Updating the Know Your Chances Website to Include Smoking Status as a Risk Factor for Mortality Estimates
Source: JAMA Netw Open. 2023 Jun 8;6(6):e2317351. doi: 10.1001/jamanetworkopen.2023.17351 (PMC10251216; doi:10.1001/jamanetworkopen.2023.17351)
Supplement: Supplement 1. — eMethods 1. Double Decrement Lifetable eMethods 2. Estimating the RRs of Dying by Smoking Status From the NHIS-LMF Cohort Using Cox Proportional Hazards Model eMethods 3. Counterintuitive Competing Risks Example eMethods 4. Comparison Between the RRs Obtained From NHIS-LMF Cohort vs Combined Cohort for Selected Causes of Death eTable 1. Causes of Death Used on Know Your Chances Website by Association With Smoking Status and ICD-10 Codes eTable 2. Estimated Relative Risks (RRs) and Their Corresponding 95% CIs for All-Cause Mortality and 32 Smoking-Related Causes of Death eTable 3. Relative Risks (RRs) for Complement Causes of Death (ie, All-Cause Minus the Cause of Interest) for 10 Causes of Deaths From NHIS-LMF eTable 4. Chance of Dying in the Next 10 Years by Smoking Status for Black Men eTable 5. Chance of Dying in the Next 10 Years by Smoking Status for White Men eTable 6. Chance of Dying in the Next 10 Years by Smoking Status for White Women eFigure 1. The Effect of Smoking Status on the Order of the Top 5 Causes of Death and Absolute Risk of Death Over 10 Years for a 60-Year-Old White Man eFigure 2. Overview of Computational Details for Partitioning Mortality Rates by Smoking Status for Smoking-Related and Non–Smoking-Related Causes of Death eReferences [file jamanetwopen-e2317351-s001.pdf]

Supplementary Online Content

Woloshin S, Landsman V, Miller DG, Byrne J, Graubard BI, Feuer EJ. Updating the Know Your Chances website to include smoking status as a risk factor for mortality estimates. *JAMA Netw Open*. 2023;6(6):e2317351. doi:10.1001/jamanetworkopen.2023.17351

eMethods 1. Double Decrement Lifetable

eMethods 2. Estimating the RRs of Dying by Smoking Status From the NHIS-LMF Cohort Using Cox Proportional Hazards Model

eMethods 3. Counterintuitive Competing Risks Example

eMethods 4. Comparison Between the RRs Obtained From NHIS-LMF Cohort vs Combined Cohort for Selected Causes of Death

eTable 1. Causes of Death Used on Know Your Chances Website by Association With Smoking Status and *ICD-10* Codes

eTable 2. Estimated Relative Risks (RRs) and Their Corresponding 95% CIs for All-Cause Mortality and 32 Smoking-Related Causes of Death

eTable 3. Relative Risks (RRs) for Complement Causes of Death (ie, All-Cause Minus the Cause of Interest) for 10 Causes of Deaths From NHIS-LMF

eTable 4. Chance of Dying in the Next 10 Years by Smoking Status for Black Men

eTable 5. Chance of Dying in the Next 10 Years by Smoking Status for White Men

eTable 6. Chance of Dying in the Next 10 Years by Smoking Status for White Women

eFigure 1. The Effect of Smoking Status on the Order of the Top 5 Causes of Death and Absolute Risk of Death Over 10 Years for a 60-Year-Old White Man

eFigure 2. Overview of Computational Details for Partitioning Mortality Rates by Smoking Status for Smoking-Related and Non–Smoking-Related Causes of Death

eReferences

This supplementary material has been provided by the authors to give readers additional information about their work.

© 2023 Woloshin S et al. *JAMA Network Open*.

eMethods 1. Double Decrement Lifetable

DEVCAN calculations utilize a double decrement lifetable which starts with a hypothetical cohort of 10,000,000 newborn individual. For each age interval the lifetable decrements the number who die from cause D, and the number who die from all other causes, excluding D (O). These deaths are then iteratively subtracted from the number alive at the beginning of the age interval to estimate the number alive at the start of the next age interval. The calculation can be terminated at any specific age or the final age interval can be open-ended (e.g., age 85 and above). In an open-ended interval all individuals alive at the start of the interval die of either cause D or O. The age conditional probability of dying of cause D by age *y* given an individual alive at age *x* is computed using the lifetable as the number who die of cause D from ages *x* to *y* divided by the number alive at age *x*. The lifetime probability of dying from cause D is computed as the total number who die of cause D across all ages divided by 10,000,000. The input data are the age specific mortality rates for causes D and O. To create reasonably stable inputs, the age intervals for the inputs are usually grouped as (<1, 1-4, 5-9, …, 80-84, 85+) or sometimes up to 95+. However, internally, all calculations are done in half year age increments, with the input data smoothed using a piecewise mid-age group joinpoint (PMAJ) method.[1-3]

© 2023 Woloshin S et al. *JAMA Network Open*.

eMethods 2. Estimating the RRs of Dying by Smoking Status From the NHIS-LMF Cohort Using Cox Proportional Hazards Model

The NHIS-LMF cohort of surveyed individuals of age 40 or older (N =529,804) was assembled and used for the estimation of the RRs for 10 (out of 32) smoking-related causes of deaths (D), for which at least 50 deaths were observed for each category of smoking (see **eTable 2**). The RRs for the corresponding complement causes (O) as well as all-cause mortality were also estimated from this cohort. We estimated the RRs in the presence of competing causes (technically called crude sub-distribution RRs)[4] by fitting Cox proportional hazards regression models with modified risk sets to the data. Crude RRs account for deaths from competing causes by constructing the modified risk sets so that they include both individuals who survive and those who die from a competing cause of death.[5] In general, because some people die of other causes before they can die of cause D, crude RRs are closer to 1 than net (sometimes called pure) RRs, that account for death from a given cause D when all other causes of death are eliminated. These crude RRs are used as input into equations (see Boxes (a) and (b) in the main manuscript) to compute the mortality rates by smoking status, which are then used as input into DevCan. For the 10 causes, we were able to estimate the crude RRs from the NHIS-LMF. For the remaining 22 RRs where available we used published net estimates (pooled data from five cohorts of US volunteers, i.e., the 'combined cohort'[6]). RRs for five of these 22 causes of death were not available from the combined cohort.[6] RRs for abdominal aortic aneurysm, aortic aneurysm outside the aorta, and peripheral arterial disease were estimated from only the NIH-AARP[7] and CPS-II[8] Nutrition cohorts in the combined cohort[6] (comprising ~ 70% of the combined cohort[6]), and using the same methodology (Christina Newton, MPH, American Cancer Association, email communication, August 25, 2016). The RR for cervical cancer was obtained using the full CPS-II cohort[9]. Because estimates were unstable for laryngeal cancer for women due to the small number or deaths, we combined the RRs for men and women from the combined cohort[6], weighting them by the inverse of their variances. The RRs from the combined cohort[6] were available for ages $\geq$55 and were applied without further adjustments to ages 40-84 in our calculations.

For the 10 causes, for which the NHIS-LMF data was available, both crude and net RR estimates were obtained and are presented in **eMethods 4 table**, which allowed us to compute the ratios of crude to net RRs for current and former smokers. These ratios appeared to be fairly consistent within smoking categories across the 10 causes for men and women. We averaged these 10 ratios to obtain a rough `correction' factor (i.e., the ratio of crude to net estimates) of 0.865 for current and 0.943 for former smokers. These factors were applied to extrapolate the crude RRs for the 22 causes for which only net published RRs were available (i.e., by multiplying the net estimates by the correction factor). The results of this sensitivity analysis showed about a 13% decrease in the risk of death for current and 5% decrease for former smokers ages 60-80, which corresponds to an average absolute risk difference of 0.05 and 0.01 percentage point for current and former smokers respectively, resulting in a slight attenuation of the risk of smoking.

© 2023 Woloshin S et al. *JAMA Network Open*.

For 10 causes of death estimated from the NHIS-LMF data were obtained for ages 40-95 and were applied without further adjustments to ages 40-85.

Time-scale Time-on-study, defined as number of days from the start of follow-up (i.e., interview date) to the end of follow-up, was used as the time-scale in the Cox regression models. The estimation of the crude RRs using the Fine and Gray approach from the NHIS-LMF data, in which censoring results from administrative loss-to-follow-up only, is simplified by “delaying” the end of follow-up to the end of study for those who experienced the competing risk event.[4] Participant’s age at the start of follow-up was used as a covariate to adjust for the effect of age on mortality. This approach is justified for Cox regression models, in which the shape of the survival distribution is Gompertz, which is generally used to model human survival.[10]

Age intervals The RRs for all-cause mortality as well as complement causes were obtained for four age groups: [40-55), [55-65), [65-75) and [75-95). For example, a 54 years-old person with 9 years of follow-up, contributed 1 year of their follow-up time to the age interval [40-55) and 8 years of their follow-up time to the age interval [55-65). The RRs for lung cancer were obtained for three age groups [40-60), [60-75), [75-95) and the RRs for the remaining 9 causes were obtained for one age group [40-95) due to insufficient numbers of deaths in the smaller age groups.

Length of follow-up Since participants enter the cohort at variable times, i.e., time of the NHIS interview, the most recent surveyed individuals will have significantly shorter time of follow-up compared to the individuals who were surveyed in the earlier years. Moreover, smoking habits of current smokers tend to change over time (i.e., they may become former smokers) Therefore, current smokers whose follow-up was longer than 10 years, were considered censored even if the death event occurred after the period of 10 years.

Covariates included in the Cox regression Smoking status (current, former, never), race (white, black, all races), and age at the start of follow-up were included as covariates in the regression models. Never smokers and white race were selected as reference levels for smoking status and race, respectively. Separate regression models were fitted for males and females.

eMethods 3. Counterintuitive Competing Risks Example

In many instances the results of a competing risks analysis can be quite counterintuitive. For example, the relative risk of dying of colorectal cancer for males is 1.31 for a current smoker, and 1.15 for a former smoker, relative to a never smoker. Despite these relative risks, the chance of dying of colorectal cancer from age 65 to 85 is highest for former smokers (1.09%), next highest for never smokers (1.02%), and lowest for current smokers (0.97%). Even more puzzling is that the risks of dying of colorectal cancer line up as expected from age 65 to 70 (0.21%, 0.24%, 0.26%), 70 to 75 (0.27%, 0.31%, 0.32%), 75 to 80 (0.38%, 0,40%, 0.42%), and 80 to 85 (0.34%, 0.39%, 0.41%) for never, former, and current smokers respectively. This type of seeming contradiction occurs when the relative risk for the specified cause is moderate, but the relative risks for other causes is significantly larger. In this case the relative risk for causes other

© 2023 Woloshin S et al. *JAMA Network Open*.

than colorectal cancer is 2.08, 3.17, 3.13, and 2.19 for current smokers and 1.12, 1.30, 1.43 and 1.32 for former smokers aged 40-55, 55-65, 65-75, and 75-85 respectively relative to a never smoker. The table below presents the portion of the DevCan lifetable from ages 65 to 85. The risk from any age *x* through age *y* is computed as the cumulative number of deaths of a specified cause from ages *x* to *y* divided by the number alive at the start of age interval *x*. Because the risk of dying of other causes is much larger for current smokers than for never smokers, the risk set (i.e., # alive at the beginning of each interval) is depleted much faster for current smokers. Even though in each age interval the interval risk is larger for current smokers, that risk is applied to a smaller number of individuals who are alive at the start of each successive interval, and thus yields fewer deaths. It takes several intervals of this cumulative depletion of the risk set to actually reverse the order of the risk estimates. As shown in **eMethods 3 table**, the risk of dying of colorectal cancer from ages 65-75 is in the expected order (0.47%, 0.52%, 0.53%) for never, former, and current smokers respectively. From ages 65-80 former smokers have the highest risk (0.84%), followed by current smokers (0.79%), and never smokers (0.77%). As described earlier, the risk from ages 65 to 85 shows even more of a reversal with the highest risk for former smokers, followed by never smokers, with current smokers having the smallest risk.

eMethods 3 table. Portion of DEVCAN lifetable from ages 65 to 85 for never, former and current male smokers (all races combined). This table highlights the counterintuitive effect that accounting for competing risks can have on the chance of cancer death when the relative risk for the specified cause is moderate (here colorectal cancer), but the relative risks for other causes are significantly larger.

	Never Smoker			Former Smoker			Current Smoker		
Age Interval	Total Alive At Beginning Of Interval	Number Who Die From Cancer This Interval	Number Who Die From Other Causes This Interval	Total Alive At Beginning Of Interval	Number Who Die From Cancer This Interval	Number Who Die From Other Causes This Interval	Total Alive At Beginning Of Interval	Number Who Die From Cancer This Interval	Number Who Die From Other Causes This Interval
65 <= Age < 70	8,382,865	17,690	486,182	8,145,158	19,537	660,707	6,811,583	17,686	1,162,738
70 <= Age < 75	7,878,993	21,325	700,050	7,464,914	22,816	913,186	5,631,159	18,243	1,342,389
75 <= Age < 80	7,157,617	25,563	1,049,879	6,528,912	26,162	1,235,057	4,270,526	18,105	1,284,928
80 <= Age < 85	6,082,176	20,970	947,981	5,267,693	20,359	1,051,607	2,967,493	12,161	920,607
		% Dying CRC	% Dying OC		% Dying CRC	% Dying OC		% Dying CRC	% Dying OC
65 <= Age < 70		0.21%	5.80%		0.24%	8.11%		0.26%	17.07%
70 <= Age < 75		0.27%	8.89%		0.31%	12.23%		0.32%	23.84%
75 <= Age < 80		0.36%	14.67%		0.40%	18.92%		0.42%	30.09%
80 <= Age < 85		0.34%	15.59%		0.39%	19.96%		0.41%	31.02%
65 <= Age < 75		0.47%	14.15%		0.52%	19.32%		0.53%	36.78%
65 <= Age < 80		0.77%	26.67%		0.84%	34.49%		0.79%	55.64%
65 <= Age < 85		1.02%	37.98%		1.09%	47.40%		0.97%	69.16%

© 2023 Woloshin S et al. *JAMA Network Open*.

eMethods 4. Comparison Between the RRs Obtained From NHIS-LMF Cohort vs Combined Cohort for Selected Causes of Death

We also used the NHIS-LMF cohort to estimate the RRs for several selected causes of death using a methodology closely mimicking the one used by Carter et al.[6], where we try to match their age groups and covariates. The selected causes of deaths were defined with strict correspondence to the definitions in by Carter et. al.[6] In addition, since the RRs published by Carter et al.[6] do not account for competing events (i.e., net RRs), we estimated both crude and net RRs using the NHIS-LMF data. These calculations are viewed as a sensitivity analysis to show the change in magnitude of the RRs for crude versus net analyses within the NHIS-LMF cohort and compared with the crude HRs from the combined cohort data. The largest differences between NHIS-LMF and the combined cohort were for lung cancer and COPD.

eMethods 4 table. Crude and net RRs and their corresponding 95% confidence intervals, CIs estimated from the NHIS-LMF cohort and net RRs and their corresponding 95% confidence intervals, CIs from Carter et. al.[6] for all-cause mortality and mortality from 11 selected causes of deaths (that closely match the 10 causes of death in **eTable 2** for which the NHIS-LMF crude rates have been used in the calculations – high blood pressure (I10-I15) being split into two causes hypertensive heart disease (I11) and essential and secondary hypertension (I10, I15)). T/C/F = **T**otal/**C**urrent smokers/**F**ormer smokers. Age interval: [55; 95) for NHIS – LMF; 55+ for combined cohort.

Cause of death and ICD-10 as used in Carter et al.[6]	Data Source	Women			Men		
		Number of deaths (T/C/F)	Current RR (95% CI)	Former RR (95% CI)	Number of deaths (T/C/F)	Current RR (95% CI)	Former RR (95% CI)
All-cause	NHIS – LMF	19,618/ 3,955/ 5,237	2.70 (2.58, 2.82)	1.54 (1.48, 1.60)	16,985/ 4,537/ 7,810	2.57 (2.44, 2.70)	1.35 (1.29, 1.41)
	Combined cohort	77,429/ 8,150/ 37,493	2.8 (2.7, 2.9)	1.5 (1.5, 1.6)	103,948/ 8,325/ 70,760	2.8 (2.8, 2.9)	1.5 (1.5, 1.5)
Colon and rectum cancer (C18-C20)	NHIS – LMF (crude)	429/ 65/ 115	1.55 (1.11, 2.17)	1.38 (1.08, 1.76)	415/ 90/ 189	1.24 (0.91, 1.69)	1.11 (0.85, 1.45)
	NHIS – LMF (net)		1.81 (1.29, 2.54)	1.48 (1.16, 1.89)		1.40 (1.03, 1.91)	1.15 (0.89, 1.50)
	Combined cohort (net)	2,199/ 174/ 1,009	1.6 (1.4, 1.9)	1.2 (1.1, 1.3)	2,745/ 160/ 1,832	1.4 (1.2, 1.7)	1.2 (1.1, 1.3)

© 2023 Woloshin S et al. *JAMA Network Open*.

Cause of death and ICD-10 as used in Carter et al.[6]	Data Source	Women			Men		
		Number of deaths (T/C/F)	Current RR (95% CI)	Former RR (95% CI)	Number of deaths (T/C/F)	Current RR (95% CI)	Former RR (95% CI)
Liver (C22)	NHIS – LMF (crude)	115/ 27/ 31	1.83 (1.06, 3.16)	1.84 (1.09, 3.10)	165/ 52/ 67	2.01 (1.20, 3.37)	1.36 (0.83, 2.22)
	NHIS – LMF (net)		2.01 (1.15, 3.50)	1.92 (1.14, 3.25)		2.25 (1.34, 3.75)	1.40 (0.86, 2.28)
	Combined cohort (net)	465/ 40/ 197	1.8 (1.3, 2.5)	1.1 (0.9, 1.4)	952/ 74/ 650	2.3 (1.8, 3.0)	1.5 (1.3, 1.7)
Pancreatic cancer (C25)	NHIS – LMF (crude)	346/ 75/ 83	1.90 (1.36, 2.65)	1.28 (0.94, 1.74)	280/ 68/ 121	1.46 (0.99, 2.14)	1.07 (0.78, 1.48)
	NHIS – LMF (net)		2.16 (1.54, 3.02)	1.36 (1.00, 1.85)		1.64 (1.11, 2.42)	1.11 (0.81, 1.52)
	Combined cohort (net)	2,093/ 184/ 961	1.9 (1.6, 2.2)	1.2 (1.1, 1.4)	2,369/ 153/ 1,469	1.6 (1.4, 1.9)	1.0 (0.9, 1.1)
Trachea, lung, and bronchus cancer (C33-C34)	NHIS – LMF (crude)	1,306/ 671/ 425	16.81 (13.88, 20.35)	5.51 (4.51, 6.73)	1,436/ 759/ 564	13.02 (10.18, 16.64)	3.56 (2.79, 4.55)
	NHIS – LMF (net)		18.82 (15.51, 22.83)	5.81 (4.75, 7.11)		14.40 (11.24, 18.44)	3.68 (2.89, 4.70)
	Combined cohort (net)	6,715/ 1,872/ 4,108	22.9 (21.0, 25.0)	6.8 (6.2, 7.3)	8,790/ 1,754/ 6,556	25.3 (22.8, 28.1)	6.8 (6.2, 7.5)
Diabetes (E10-E14)	NHIS – LMF (crude)	701/ 104/ 179	1.19 (0.91, 1.56)	1.25 (1.02, 1.54)	582/ 138/ 257	1.41 (1.06, 1.87)	1.13 (0.90, 1.42)
	NHIS – LMF (net)		1.36 (1.04, 1.78)	1.34 (1.09, 1.64)		1.63 (1.22, 2.16)	1.18 (0.94, 1.48)
	Combined cohort (net)	1,552/ 110/ 699	1.5 (1.3, 1.9)	1.4 (1.2, 1.5)	2,794/ 142/ 1,923	1.6 (1.3, 1.9)	1.4 (1.3, 1.6)

© 2023 Woloshin S et al. *JAMA Network Open*.

Cause of death and ICD-10 as used in Carter et al.[6]	Data Source	Women			Men		
		Number of deaths (T/C/F)	Current RR (95% CI)	Former RR (95% CI)	Number of deaths (T/C/F)	Current RR (95% CI)	Former RR (95% CI)
Ischemic heart disease (I20-I25)	NHIS – LMF (crude)	3,135/ 555/ 801	2.21 (1.96, 2.49)	1.33 (1.20, 1.46)	3,418/ 843/ 1,591	2.03 (1.83, 2.26)	1.21 (1.10, 1.33)
	NHIS – LMF (net)		2.60 (2.30, 2.93)	1.44 (1.30, 1.58)		2.32 (2.09, 2.58)	1.27 (1.15, 1.40)
	Combined cohort (net)	9,746/ 1,014/ 4,613	3.0 (2.8, 3.2)	1.6 (1.5, 1.7)	20,185/ 1,522/ 13,716	2.6 (2.4, 2.7)	1.5 (1.4, 1.5)
Total stroke (I60-I69)	NHIS – LMF (crude)	1,357/ 183/ 294	1.55 (1.26, 1.91)	0.88 (0.76, 1.03)	823/ 143/ 392	1.37 (1.08, 1.73)	1.13 (0.94, 1.37)
	NHIS – LMF (net)		1.86 (1.51, 2.30)	0.97 (0.83, 1.13)		1.62 (1.28, 2.05)	1.20 (0.99, 1.45)
	Combined cohort (net)	4,966/ 385/ 2,146	2.1 (1.8, 2.3)	1.2 (1.1, 1.3)	4,855/ 279/ 3,177	1.9 (1.7, 2.2)	1.2 (1.1, 1.3)
Hypertensive heart disease (I11)	NHIS – LMF (crude)	261/ 45/ 55	1.94 (1.24, 3.04)	1.09 (0.75, 1.58)	192/ 62/ 79	2.73 (1.74, 4.29)	1.39 (0.91, 2.13)
	NHIS – LMF (net)		2.30 (1.48, 3.58)	1.18 (0.81, 1.71)		3.12 (1.99, 4.89)	1.45 (0.95, 2.20)
	Combined cohort (net)	521/ 45/ 232	1.9 (1.4, 2.7)	1.3 (1.1, 1.6)	776/ 75/ 508	2.9 (2.2, 3.9)	1.3 (1.1, 1.6)
Essential and secondary hypertension (I10, I15)	NHIS – LMF (crude)	173/ 25/ 42	2.24 (1.35, 3.71)	1.22 (0.80, 1.87)	82/ 28/ 28	2.32 (1.22, 4.41)	0.86 (0.45, 1.67)
	NHIS – LMF (net)		2.72 (1.64, 4.51)	1.33 (0.87, 2.04)		2.73 (1.41, 5.30)	0.91 (0.47, 1.77)
	Combined cohort (net)	499/ 48/ 202	2.4 (1.7, 3.4)	1.1 (0.9, 1.3)	619/ 49/ 395	2.6 (1.9, 3.6)	1.2 (1.0, 1.4)

© 2023 Woloshin S et al. *JAMA Network Open*.

Cause of death and ICD-10 as used in Carter et al.[6]	Data Source	Women			Men		
		Number of deaths (T/C/F)	Current RR (95% CI)	Former RR (95% CI)	Number of deaths (T/C/F)	Current RR (95% CI)	Former RR (95% CI)
Pneumonia, influenza, and tuberculosis (J10-J18, A16-A19)	NHIS – LMF (crude)	431/ 68/ 105	1.86 (1.32, 2.64)	1.13 (0.86, 1.49)	337/ 65/ 171	1.59 (1.09, 2.32)	1.26 (0.95, 1.67)
	NHIS – LMF (net)		2.29 (1.61, 3.25)	1.25 (0.95, 1.64)		1.91 (1.31, 2.78)	1.34 (1.02, 1.77)
	Combined cohort (net)	1,520/ 100/ 697	1.9 (1.6, 2.4)	1.3 (1.2, 1.5)	1,894/ 87/ 1,320	2.0 (1.6, 2.6)	1.5 (1.4, 1.7)
COPD (J40-J44)	NHIS – LMF (crude)	1,272/ 552/ 504	16.76 (13.68, 20.54)	6.96 (5.69, 8.51)	1,114/ 445/ 565	11.29 (8.64, 14.76)	4.01 (3.12, 5.16)
	NHIS – LMF (net)		19.53 (15.95, 23.90)	7.49 (6.13, 9.16)		13.13 (10.05, 17.17)	4.21 (3.27, 5.41)
	Combined cohort (net)	4,239/ 941/ 2,888	25.0 (22.2, 28.1)	9.2 (8.2, 10.2)	5,007/ 825/ 3,923	27.8 (24.1, 32.0)	7.5 (6.7, 8.6)

© 2023 Woloshin S et al. *JAMA Network Open*.

eTable 1. Causes of Death Used on Know Your Chances Website by Association With Smoking Status and *ICD-10* Codes

Cause of Death	Smoking-related cause	ICD-10
**All-cause**	Yes	A00-Y89
Abdominal Aortic Aneurysm	Yes	I71.3, I71.4
Acute Myeloid Leukemia	Yes	C92.0
Aortic Aneurysm Outside Abdomen	Yes	I71.0-I71.2, I71.5-I71.9
Breast Cancer	Yes	C50
Cervix Cancer	Yes	C53
Chronic Liver Disease and Cirrhosis	Yes	K70, K73-K74
Colon and Rectum	Yes	C18-C21, C26.0
COPD	Yes	J40-J44, J47
Coronary Heart Disease	Yes	I20-I25, I42.0, I42.8, I42.9, I44-I46, I50
Diabetes	Yes	E10-E14
Diffuse Atherosclerosis	Yes	I70
Esophagus Cancer	Yes	C15
Heart Lining Disease	Yes	I30-I32
Heart Rhythm Disease (Atrial fibrillation/flutter)	Yes	I47-I49
Heart Valve Disease	Yes	I00-I02, I05-I09, I33-I39
High Blood Pressure	Yes	I10-I15
Kidney and Renal Pelvis Cancer	Yes	C64-C65
Larynx Cancer	Yes	C32
Liver Cancer	Yes	C22
Lung and Bronchus Cancer	Yes	C34
Lung Circulation Disease	Yes	I26-I28
Oral Cavity and Pharynx Cancer	Yes	C0-C14
Pancreatic Cancer	Yes	C25
Parkinson's Disease	Yes	G20
Peripheral Vascular Disease (PVD)	Yes	I739
Pneumonia/Flu	Yes	J09-J18
Prostate Cancer	Yes	C619
Stomach Cancer	Yes	C16
Stroke	Yes	I60-I69
Tuberculosis	Yes	A15-A19
Urinary Bladder Cancer	Yes	C67
Accidents	No	V01-X59, Y85-Y86
Acute Lymphocytic Leukemia	No	C91.0
Acute Monocytic Leukemia	No	C93.0
AIDS	No	B20-B24
Alzheimer's Disease	No	G30
Appendicitis	No	K35-K38
Asthma	No	J45-J46
Birth Defects	No	Q00-Q99
Brain and Other Nervous Systems	No	C70-C72
Bronchitis	No	J20-J21
Chicken Pox and Shingles	No	B01-B02
Chronic Lymphocytic Leukemia	No	C91.1

© 2023 Woloshin S et al. *JAMA Network Open*.

Cause of Death	Smoking-related cause	ICD-10
Chronic Myeloid Leukemia	No	C92.1
Complications of Health Care	No	Y40-Y84, Y88
Criminal Justice System	No	Y35, Y89.0
Enlargement of the Prostate	No	N40
Gallbladder Disease	No	K80-K82
Hernia	No	K40-K46
Hodgkin Lymphoma	No	C81
Kidney Failure	No	N00-N07, N17-N19, N25-N27
Kidney Infections	No	N10-N12, N13.6, N15.1
Lung Disease Due to Aspiration	No	J69
Lung Disease from Environmental Exposure	No	J60-J66, J68
Malaria	No	B50-B54
Malnutrition and Vitamin Deficiencies	No	E40-E64
Measles	No	B05
Melanoma	No	C43
Meningitis	No	A39, G00, G03
Mesothelioma	No	C45
Multiple Myeloma	No	C90.0, C90.2
Non-Hodgkin Lymphoma	No	C82-C85, C96.3
Other Anemia	No	D50-D54, D60-D64
Ovarian Cancer	No	C56
Polio	No	A80
Pregnancy Related	No	O00-O99
Rabies	No	A82
Salmonella Infections	No	A01-A02
Scarlet Fever and Erysipelas	No	A38, A46
Septicemia	No	A40-A41
Shigellosis and Amebiasis	No	A03, A06
Sickle Cell Anemia	No	D57
Suicide	No	U03, X60-X84, Y87.0
Syphilis	No	A50-A53
Testicular Cancer	No	C62
Tetanus	No	A34-A35
Thalassemia and Other Hereditary Anemias	No	D55-D56, D58-D59
Thyroid	No	C73
Tick Related Brain Infections	No	A83-A84, A85.2
Ulcer Disease	No	K25-K28
Uterine Cancer	No	C54-C55
Viral Hepatitis	No	B15-B19
War	No	Y36, Y89.1
Whooping Cough	No	A37

© 2023 Woloshin S et al. *JAMA Network Open*.

eTable 2. Estimated Relative Risks (RRs) and Their Corresponding 95% CIs for All-Cause Mortality and 32 Smoking-Related Causes of Death

				Women				Men			
				Current		Former		Current		Former	
**Cause of Death**	**ICD-10 codes**	**Data source for RR**	**Age interval**	**RR**	**95% CI**	**RR**	**95% CI**	**RR**	**95% CI**	**RR**	**95% CI**
All-cause	A00-Y89	NHIS-LMF	[40-55)	2.15	(1.94, 2.39)	0.97	(0.83, 1.14)	2.03	(1.85, 2.24)	1.11	(0.98, 1.26)
All-cause	A00-Y89	NHIS-LMF	[55-65)	2.91	(2.65, 3.19)	1.30	(1.15, 1.46)	3.13	(2.85, 3.43)	1.30	(1.17, 1.43)
All-cause	A00-Y89	NHIS-LMF	[65-75)	2.95	(2.73, 3.20)	1.48	(1.37, 1.60)	3.10	(2.85, 3.37)	1.43	(1.32, 1.55)
All-cause	A00-Y89	NHIS-LMF	[75-95)	2.35	(2.22, 2.50)	1.34	(1.29, 1.39)	2.19	(2.04, 2.35)	1.31	(1.26, 1.37)
Abdominal aortic aneurysm	I71.3, I71.4	NIH-AARP/CPSII	55+	18.6	(10.1, 34.2)	4	(2.3, 6.7)	13.8	(9.2, 20.7)	3.3	(2.4, 4.7)
Aortic aneurysm outside abdomen	I71.0-I71.2, I71.5-I71.9	NIH-AARP/CPSII	55+	7.1	(4.7, 10.8)	1.6	(1.2, 2.3)	3.9	(2.6, 5.8)	1.7	(1.3, 2.2)
Acute myeloid leukemia	C92.0	Combined cohort	55+	1.1	(0.7, 1.7)	1.1	(0.9, 1.4)	1.9	(1.4, 2.7)	1.4	(1.2, 1.6)
Breast cancer (female)	C50	Combined cohort	55+	1.3	(1.2, 1.5)	1.2	(1.1, 1.3)	n/a	n/a	n/a	n/a
Cervix cancer	C53	Full CPSII	30+	1.5	(0.9, 2.6)	1.4	(0.8, 2.6)	n/a	n/a	n/a	n/a
Chronic liver disease and cirrhosis	K70, K73-K74	Combined cohort	55+	2.6	(2.0, 3.5)	1.2	(1.0, 1.5)	3.6	(2.8, 4.6)	1.3	(1.1, 1.6)
Colon and rectum cancer	C18-C21, C26.0	NHIS-LMF	[40-95)	1.38	(1.05, 1.81)	1.19	(0.97, 1.45)	1.31	(1.01, 1.69)	1.15	(0.95, 1.40)
COPD	J40-J44, J47	NHIS-LMF	[40-95)	14.48	(12.30, 17.06)	5.30	(4.52, 6.20)	11.00	(8.79, 13.76)	3.58	(2.92, 4.38)
Coronary heart disease	I20-I25, I42.0, I42.8, I42.9, I44-I46, I50	NHIS-LMF	[40-95)	1.98	(1.80, 2.18)	1.19	(1.11, 1.28)	2.06	(1.90, 2.23)	1.17	(1.10, 1.26)
Diabetes	E10-E14	NHIS-LMF	[40-95)	1.17	(0.93, 1.47)	1.07	(0.91, 1.26)	1.57	(1.26, 1.94)	1.19	(1.00, 1.41)
Diffuse atherosclerosis	I70	Combined cohort	55+	2.1	(1.1, 4.0)	1.6	(1.2, 2.2)	5.0	(3.2, 7.9)	1.4	(1.1, 1.9)
Esophagus cancer	C15	Combined cohort	55+	5.1	(3.5, 7.4)	2.2	(1.7, 2.9)	3.9	(3.0, 5.0)	2.6	(2.2, 3.0)
Heart lining disease	I30-I32	Combined cohort	55+	1.9	(1.7, 2.1)	1.4	(1.3, 1.5)	2.0	(1.8, 2.2)	1.3	(1.2, 1.4)
Heart rhythm disease (atrial fibrillation/flutter)	I47-I49	Combined cohort	55+	1.9	(1.7, 2.1)	1.4	(1.3, 1.5)	2.0	(1.8, 2.2)	1.3	(1.2, 1.4)
Heart valve disease	I33-I39	Combined cohort	55+	1.9	(1.7, 2.1)	1.4	(1.3, 1.5)	2.0	(1.8, 2.2)	1.3	(1.2, 1.4)
High blood pressure	I10-I15	NHIS-LMF	[40-95)	1.76	(1.36, 2.27)	1.02	(0.84, 1.24)	1.72	(1.31, 2.24)	1.04	(0.84, 1.29)
Kidney and renal pelvis cancer	C64-C65	Combined cohort	55+	1.2	(0.9, 1.8)	1.2	(1.0, 1.4)	1.8	(1.4, 2.4)	1.5	(1.3, 1.7)
Larynx cancer	C32	Combined cohort*	55+	17.4	(10.7, 28.3)	2.8	(1.8, 4.3)	17.4	(10.7, 28.3)	2.8	(1.8, 4.3)
Liver cancer	C22	NHIS-LMF	[40-95)	1.69	(1.06, 2.69)	1.16	(0.77, 1.74)	2.29	(1.55, 3.38)	1.51	(1.07, 2.14)
Lung and bronchus cancer	C34	NHIS-LMF	[40-60)	8.6	(6.02, 12.27)	1.31	(0.75, 2.27)	9.79	(6.37, 15.04)	1.59	(0.94, 2.72)
Lung and bronchus cancer	C34	NHIS-LMF	[60-75)	19.98	(14.96, 26.68)	5.53	(4.07, 7.52)	12.87	(9.47, 17.49)	2.87	(2.08, 3.95)
Lung and bronchus cancer	C34	NHIS-LMF	[75-95)	13.69	(11.04, 16.98)	4.76	(3.91, 5.80)	14.67	(10.88, 19.78)	4.39	(3.28, 5.87)

© 2023 Woloshin S et al. *JAMA Network Open*.

Cause of Death	ICD-10 codes	Data source for RR	Age interval	Women				Men			
				**Current**		**Former**		**Current**		**Former**	
				**RR**	**95% CI**	**RR**	**95% CI**	**RR**	**95% CI**	**RR**	**95% CI**
Lung circulation disease	I26-I28	Combined cohort	55+	1.9	(1.7, 2.1)	1.4	(1.3, 1.5)	2.0	(1.8, 2.2)	1.3	(1.2, 1.4)
Oral cavity and pharynx cancer	C00-C14	Combined cohort	55+	5.6	(3.7, 8.6)	2.2	(1.6, 3.1)	5.7	(4.1, 8.1)	1.7	(1.3, 2.2)
Pancreatic cancer	C25	NHIS-LMF	[40-95)	2.05	(1.53, 2.74)	1.23	(0.97, 1.57)	1.58	(1.17, 2.14)	0.99	(0.76, 1.29)
Parkinson's disease	G20	Combined cohort	55+	0.4	(0.2, 0.6)	0.8	(0.7, 0.9)	0.4	(0.3, 0.6)	0.9	(0.8, 1.0)
Peripheral vascular disease (PVD)	I73.9	NIH-AARP/CPSII	55+	6.5	(3.6, 11.5)	2.9	(1.9, 4.3)	5.4	(3.2, 9.0)	1.6	(1.1, 2.3)
Pneumonia/Flu	J09-J18	NHIS-LMF	[40-95)	1.80	(1.36, 2.39)	1.07	(0.86, 1.31)	1.39	(1.03, 1.87)	1.05	(0.83, 1.31)
Prostate cancer	C619	Combined cohort	55+	n/a	n/a	n/a	n/a	1.4	(1.2, 1.7)	1.0	(1.0, 1.1)
Stomach cancer	C16	Combined cohort	55+	1.7	(1.2, 2.5)	1.1	(0.9, 1.4)	1.9	(1.4, 2.7)	1.5	(1.2, 1.8)
Stroke	I60-I69	NHIS-LMF	[40-95)	1.52	(1.27, 1.81)	0.85	(0.76, 0.96)	1.29	(1.05, 1.57)	1.07	(0.92, 1.25)
Tuberculosis	A15-A19	Combined cohort	55+	1.9	(1.6, 2.4)	1.3	(1.2, 1.5)	2.0	(1.6, 2.6)	1.5	(1.4, 1.7)
Urinary bladder cancer	C67	Combined cohort	55+	3.9	(2.8, 5.5)	2.3	(1.8, 2.9)	3.9	(3.0, 5.1)	2.4	(2.1, 2.8)

* Too few cases in women, used a pooled estimate for men and women

Notes:

Data Sources for RRs:

NHIS-LMF – estimated by the authors from the NHIS-LMF data. Models were adjusted for age at the interview and race. Former smoker: smoked >100 cigarettes in lifetime and quit ≥ 2 years before interview

Combined cohort – see Carter, et. al.[6]

NIH-AARP/CPSII – provided by Christina Newton MPH, American Cancer Association, email communication, August 25, 2016 (includes AARP and CPSII Nutrition cohorts) - models were adjusted as in Carter, et. al.[6]

Full CPSII – see Thun, et. al.[9]

© 2023 Woloshin S et al. *JAMA Network Open*.

eTable 3. Relative Risks (RRs) for Complement Causes of Death (ie, All-Cause Minus the Cause of Interest) for 10 Causes of Deaths From NHIS-LMF

Cause of interest	Age interval	Women				Men			
		Current		Former		Current		Former	
		RR	95% CI	RR	95% CI	RR	95% CI	RR	95% CI
Colon and rectum cancer	[40, 55)	2.21	(1.99, 2.46)	0.95	(0.81, 1.12)	2.08	(1.88, 2.29)	1.12	(0.98, 1.27)
	[55, 65)	2.94	(2.67, 3.23)	1.29	(1.14, 1.45)	3.17	(2.89, 3.49)	1.3	(1.18, 1.44)
	[65, 75)	3.00	(2.77, 3.25)	1.5	(1.39, 1.63)	3.13	(2.88, 3.41)	1.43	(1.32, 1.55)
	[75, 95)	2.35	(2.21, 2.50)	1.34	(1.29, 1.39)	2.19	(2.05, 2.35)	1.31	(1.26, 1.37)
COPD	[40, 55)	2.08	(1.87, 2.31)	0.97	(0.82, 1.13)	2.01	(1.82, 2.21)	1.12	(0.98, 1.27)
	[55, 65)	2.68	(2.44, 2.95)	1.24	(1.10, 1.40)	2.95	(2.68, 3.24)	1.28	(1.15, 1.41)
	[65, 75)	2.54	(2.33, 2.77)	1.37	(1.27, 1.49)	2.75	(2.53, 3.00)	1.37	(1.26, 1.48)
	[75, 95)	1.82	(1.70, 1.95)	1.19	(1.15, 1.24)	1.78	(1.65, 1.92)	1.19	(1.14, 1.25)
Coronary heart disease	[40, 55)	2.07	(1.86, 2.32)	0.95	(0.80, 1.12)	1.93	(1.74, 2.14)	1.13	(0.98, 1.30)
	[55, 65)	2.81	(2.54, 3.12)	1.29	(1.14, 1.47)	3.15	(2.84, 3.49)	1.29	(1.15, 1.45)
	[65, 75)	3.06	(2.80, 3.34)	1.49	(1.37, 1.62)	3.07	(2.80, 3.37)	1.41	(1.29, 1.54)
	[75, 95)	2.27	(2.12, 2.43)	1.33	(1.27, 1.38)	2.07	(1.91, 2.24)	1.30	(1.24, 1.37)
Diabetes	[40, 55)	2.19	(1.97, 2.43)	0.97	(0.83, 1.14)	2.02	(1.84, 2.23)	1.09	(0.96, 1.24)
	[55, 65)	2.99	(2.72, 3.29)	1.31	(1.16, 1.47)	3.18	(2.90, 3.49)	1.31	(1.18, 1.45)
	[65, 75)	3.01	(2.78, 3.26)	1.50	(1.38, 1.63)	3.14	(2.89, 3.42)	1.43	(1.32, 1.55)
	[75, 95)	2.40	(2.26, 2.55)	1.34	(1.29, 1.40)	2.23	(2.08, 2.39)	1.32	(1.27, 1.38)
High blood pressure	[40, 55)	2.13	(1.92, 2.37)	0.96	(0.82, 1.13)	2.06	(1.87, 2.27)	1.12	(0.99, 1.28)
	[55, 65)	2.90	(2.64, 3.18)	1.30	(1.15, 1.46)	3.12	(2.84, 3.44)	1.31	(1.18, 1.45)
	[65, 75)	2.98	(2.75, 3.23)	1.49	(1.37, 1.61)	3.15	(2.90, 3.43)	1.44	(1.33, 1.56)
	[75, 95)	2.36	(2.22, 2.51)	1.35	(1.30, 1.40)	2.18	(2.04, 2.34)	1.31	(1.26, 1.36)
Liver cancer	[40, 55)	2.17	(1.95, 2.41)	0.98	(0.84, 1.15)	2.04	(1.86, 2.25)	1.12	(0.98, 1.27)
	[55, 65)	2.91	(2.65, 3.20)	1.30	(1.15, 1.46)	3.12	(2.84, 3.42)	1.30	(1.17, 1.44)
	[65, 75)	2.97	(2.74, 3.21)	1.49	(1.37, 1.61)	3.10	(2.85, 3.37)	1.42	(1.31, 1.54)
	[75, 95)	2.35	(2.22, 2.50)	1.34	(1.29, 1.39)	2.18	(2.03, 2.34)	1.31	(1.26, 1.37)
Lung and bronchus cancer	[40, 55)	1.99	(1.79, 2.23)	0.96	(0.82, 1.13)	1.92	(1.74, 2.12)	1.11	(0.97, 1.27)
	[55, 65)	2.46	(2.22, 2.72)	1.24	(1.10, 1.41)	2.8	(2.55, 3.08)	1.28	(1.16, 1.42)
	[65, 75)	2.29	(2.10, 2.50)	1.34	(1.23, 1.45)	2.49	(2.29, 2.72)	1.34	(1.24, 1.45)

© 2023 Woloshin S et al. *JAMA Network Open*.

Cause of interest	Age interval	Women				Men			
		Current		Former		Current		Former	
		RR	95% CI	RR	95% CI	RR	95% CI	RR	95% CI
	[75, 95)	1.93	(1.81, 2.06)	1.24	(1.19, 1.29)	1.67	(1.55, 1.80)	1.21	(1.16, 1.26)
Pancreatic Cancer	[40, 55)	2.15	(1.94, 2.39)	0.97	(0.82, 1.14)	2.05	(1.86, 2.25)	1.12	(0.99, 1.28)
	[55, 65)	2.90	(2.64, 3.19)	1.32	(1.17, 1.48)	3.16	(2.88, 3.47)	1.31	(1.19, 1.46)
	[65, 75)	2.97	(2.75, 3.22)	1.48	(1.37, 1.60)	3.16	(2.91, 3.44)	1.45	(1.34, 1.56)
	[75, 95)	2.35	(2.22, 2.50)	1.34	(1.29, 1.39)	2.18	(2.04, 2.34)	1.31	(1.26, 1.37)
Pneumonia/Flu	[40, 55)	2.15	(1.93, 2.38)	0.98	(0.84, 1.15)	2.04	(1.85, 2.24)	1.10	(0.96, 1.25)
	[55, 65)	2.91	(2.65, 3.20)	1.31	(1.16, 1.48)	3.12	(2.85, 3.43)	1.29	(1.17, 1.43)
	[65, 75)	2.96	(2.73, 3.20)	1.48	(1.36, 1.60)	3.13	(2.88, 3.40)	1.44	(1.33, 1.56)
	[75, 95)	2.34	(2.20, 2.48)	1.34	(1.29, 1.40)	2.19	(2.04, 2.35)	1.32	(1.26, 1.38)
Stroke	[40, 55)	2.17	(1.95, 2.41)	0.99	(0.85, 1.17)	2.06	(1.87, 2.27)	1.11	(0.97, 1.26)
	[55, 65)	2.94	(2.67, 3.22)	1.33	(1.18, 1.50)	3.14	(2.86, 3.45)	1.29	(1.17, 1.43)
	[65, 75)	2.99	(2.76, 3.24)	1.50	(1.39, 1.63)	3.14	(2.89, 3.43)	1.45	(1.33, 1.57)
	[75, 95)	2.37	(2.23, 2.53)	1.38	(1.33, 1.44)	2.25	(2.10, 2.41)	1.32	(1.26, 1.38)

© 2023 Woloshin S et al. *JAMA Network Open*.

eTable 4. Chance of Dying in the Next 10 Years by Smoking Status for Black Men

Custom Chart for Men, Black, Ages 30-70 Years
The numbers in each age column tell you the percent of men who will die in the next 10 years from...

Cause of Death	Smoking Status	Risk Interval (Start Age to End Age)								
		30 to <40	35 to <45	40 to <50	45 to <55	50 to <60	55 to <65	60 to <70	65 to <75	70 to <80
**All Causes***	**Never**	**2.9%**	**3.2%**	**3.8%**	**5.2%**	**6.9%**	**9.6%**	**13.6%**	**18.8%**	**27.1%**
	**Current**	**3.1%**	**4.8%**	**7.4%**	**10.8%**	**17.4%**	**26.7%**	**36.5%**	**46.4%**	**54.9%**
**Vascular Disease**										
Abdominal Aortic Aneurysm	Never	<	<	<	<	<	<	<	<	<
	Current	<	<	<	<	<	<	<	0.1%	0.1%
Coronary Heart Disease	Never	0.2%	0.3%	0.5%	0.9%	1.3%	2.0%	2.8%	4.0%	6.0%
	Current	0.3%	0.7%	1.3%	2.1%	3.4%	5.0%	6.8%	8.5%	9.7%
Heart Failure	Never	<	<	0.1%	0.1%	0.2%	0.2%	0.4%	0.5%	0.8%
	Current	<	0.1%	0.1%	0.2%	0.3%	0.4%	0.6%	0.9%	1.3%
High Blood Pressure [a]	Never	0.1%	0.2%	0.3%	0.4%	0.6%	0.8%	1.0%	1.3%	1.6%
	Current	0.1%	0.3%	0.5%	0.7%	0.9%	1.3%	1.5%	1.8%	2.2%
Stroke	Never	0.1%	0.1%	0.2%	0.3%	0.4%	0.7%	1.0%	1.5%	2.2%
	Current	0.1%	0.1%	0.2%	0.3%	0.5%	0.8%	1.2%	1.6%	2.2%
**Cancer**										
Colon and Rectum	Never	<	0.1%	0.1%	0.2%	0.3%	0.4%	0.5%	0.7%	0.8%
	Current	<	0.1%	0.1%	0.2%	0.3%	0.5%	0.6%	0.7%	0.9%
Lung and Bronchus	Never	<	<	<	0.1%	0.1%	0.2%	0.4%	0.6%	0.7%
	Current	<	0.1%	0.2%	0.6%	1.4%	2.6%	4.2%	6.0%	7.4%
Pancreas	Never	<	<	<	0.1%	0.2%	0.3%	0.4%	0.5%	0.7%
	Current	<	<	0.1%	0.1%	0.2%	0.4%	0.6%	0.7%	0.8%
Prostate	Never	<	<	<	<	0.1%	0.3%	0.6%	1.1%	1.7%
	Current	<	<	<	0.1%	0.2%	0.4%	0.7%	1.2%	1.8%
**Lung Disease**										
COPD	Never	<	<	<	<	<	0.1%	0.2%	0.3%	0.5%
	Current	<	<	0.1%	0.2%	0.5%	1.0%	1.8%	2.9%	4.2%
**Infection**										
AIDS	Never	0.1%	0.1%	0.1%	0.2%	0.2%	0.3%	0.2%	0.2%	0.1%
	Current	0.1%	0.1%	0.1%	0.2%	0.2%	0.2%	0.2%	0.2%	0.1%
Pneumonia/Flu [a]	Never	<	<	0.1%	0.1%	0.1%	0.2%	0.3%	0.5%	0.7%
	Current	<	<	0.1%	0.1%	0.2%	0.2%	0.4%	0.5%	0.7%
**Accidents and Injury**										
Accidents	Never	0.7%	0.8%	0.8%	0.9%	1.0%	1.1%	1.1%	0.9%	0.9%
	Current	0.7%	0.8%	0.8%	0.9%	1.0%	1.0%	0.9%	0.8%	0.7%
Homicide	Never	0.7%	0.5%	0.4%	0.3%	0.2%	0.2%	0.1%	0.1%	0.1%
	Current	0.7%	0.5%	0.4%	0.3%	0.2%	0.2%	0.1%	0.1%	0.1%
Suicide	Never	0.2%	0.1%	0.1%	0.1%	0.1%	0.1%	0.1%	0.1%	0.1%
	Current	0.2%	0.1%	0.1%	0.1%	0.1%	0.1%	0.1%	0.1%	0.1%
**Diabetes [a]**	**Never**	**0.1%**	**0.1%**	**0.2%**	**0.3%**	**0.4%**	**0.6%**	**0.9%**	**1.3%**	**1.6%**
	**Current**	**0.1%**	**0.2%**	**0.3%**	**0.4%**	**0.6%**	**0.9%**	**1.3%**	**1.7%**	**2.0%**
**Neurological Disease**										
Alzheimers Disease	Never	<	<	<	<	<	<	0.1%	0.3%	0.7%
	Current	<	<	<	<	<	<	0.1%	0.2%	0.6%
Parkinsons Disease	Never	<	<	<	<	<	<	0.1%	0.2%	0.4%
	Current	<	<	<	<	<	<	<	0.1%	0.1%

Tailored charts can be created on the Know Your Chances website (<URL here>)

© 2023 Woloshin S et al. *JAMA Network Open*.

eTable 5. Chance of Dying in the Next 10 Years by Smoking Status for White Men

Custom Chart for Men, White, Ages 30-70 Years
The numbers in each age column tell you the percent of men who will die in the next 10 years from...

Cause of Death	Smoking Status	Risk Interval (Start Age to End Age)								
		30 to <40	35 to <45	40 to <50	45 to <55	50 to <60	55 to <65	60 to <70	65 to <75	70 to <80
**All Causes***	**Never**	**2.0%**	**2.2%**	**2.6%**	**3.7%**	**5.0%**	**6.9%**	**9.7%**	**14.3%**	**22.7%**
	**Current**	**2.1%**	**3.3%**	**5.1%**	**7.8%**	**12.8%**	**19.7%**	**27.3%**	**37.0%**	**47.4%**
**Vascular Disease**										
Abdominal Aortic Aneurysm	Never	<	<	<	<	<	<	<	<	<
	Current	<	<	<	<	<	<	0.1%	0.1%	0.2%
Coronary Heart Disease	Never	0.1%	0.2%	0.3%	0.6%	0.9%	1.4%	2.0%	2.9%	4.9%
	Current	0.1%	0.3%	0.8%	1.4%	2.3%	3.6%	5.0%	6.6%	8.1%
Heart Failure	Never	<	<	<	<	0.1%	0.1%	0.2%	0.3%	0.6%
	Current	<	<	<	0.1%	0.1%	0.2%	0.3%	0.6%	1.0%
High Blood Pressure [a]	Never	<	0.1%	0.1%	0.1%	0.2%	0.3%	0.4%	0.5%	0.6%
	Current	<	0.1%	0.1%	0.2%	0.3%	0.5%	0.6%	0.7%	0.9%
Stroke	Never	<	<	0.1%	0.1%	0.2%	0.3%	0.5%	0.7%	1.3%
	Current	<	<	0.1%	0.1%	0.2%	0.3%	0.5%	0.8%	1.3%
**Cancer**										
Colon and Rectum	Never	<	<	0.1%	0.1%	0.2%	0.3%	0.3%	0.4%	0.6%
	Current	<	<	0.1%	0.2%	0.2%	0.3%	0.4%	0.5%	0.6%
Lung and Bronchus	Never	<	<	<	0.1%	0.1%	0.2%	0.3%	0.5%	0.7%
	Current	<	0.1%	0.2%	0.5%	1.3%	2.4%	3.8%	5.8%	7.7%
Pancreas	Never	<	<	<	0.1%	0.1%	0.2%	0.3%	0.5%	0.6%
	Current	<	<	<	0.1%	0.2%	0.3%	0.5%	0.7%	0.8%
Prostate	Never	<	<	<	<	<	0.1%	0.2%	0.4%	0.8%
	Current	<	<	<	<	0.1%	0.1%	0.3%	0.5%	0.9%
**Lung Disease**										
COPD	Never	<	<	<	<	0.1%	0.1%	0.2%	0.4%	0.7%
	Current	<	<	0.1%	0.2%	0.6%	1.3%	2.3%	4.0%	6.4%
**Infection**										
AIDS	Never	<	<	<	<	<	<	<	<	<
	Current	<	<	<	<	<	<	<	<	<
Pneumonia/Flu [a]	Never	<	<	<	<	0.1%	0.1%	0.2%	0.3%	0.5%
	Current	<	<	<	0.1%	0.1%	0.2%	0.2%	0.4%	0.6%
**Accidents and Injury**										
Accidents	Never	0.9%	0.8%	0.8%	0.8%	0.8%	0.7%	0.7%	0.7%	0.9%
	Current	0.9%	0.8%	0.8%	0.8%	0.8%	0.7%	0.6%	0.6%	0.7%
Homicide	Never	0.1%	0.1%	0.1%	0.1%	<	<	<	<	<
	Current	0.1%	0.1%	0.1%	0.1%	<	<	<	<	<
Suicide	Never	0.3%	0.3%	0.3%	0.3%	0.3%	0.3%	0.3%	0.3%	0.3%
	Current	0.3%	0.3%	0.3%	0.3%	0.3%	0.3%	0.3%	0.3%	0.3%
**Diabetes [a]**	**Never**	<	**0.1%**	**0.1%**	**0.1%**	**0.2%**	**0.3%**	**0.5%**	**0.7%**	**0.9%**
	**Current**	<	**0.1%**	**0.1%**	**0.2%**	**0.3%**	**0.5%**	**0.7%**	**0.9%**	**1.2%**
**Neurological Disease**										
Alzheimers Disease	Never	<	<	<	<	<	<	0.1%	0.3%	0.7%
	Current	<	<	<	<	<	<	0.1%	0.2%	0.6%
Parkinsons Disease	Never	<	<	<	<	<	<	0.1%	0.3%	0.7%
	Current	<	<	<	<	<	<	<	0.1%	0.2%

Tailored charts can be created on the Know Your Chances website (<URL here>)

© 2023 Woloshin S et al. *JAMA Network Open*.

eTable 6. Chance of Dying in the Next 10 Years by Smoking Status for White Women

Custom Chart for Women, White, Ages 30-70 Years
The numbers in each age column tell you the percent of women who will die in the next 10 years from...

Cause of Death	Smoking Status	Risk Interval (Start Age to End Age) 30 to <40	35 to <45	40 to <50	45 to <55	50 to <60	55 to <65	60 to <70	65 to <75	70 to <80
**All Causes***	**Never**	**1.0%**	**1.2%**	**1.6%**	**2.4%**	**3.3%**	**4.6%**	**6.7%**	**10.5%**	**17.3%**
	**Current**	**1.1%**	**2.0%**	**3.3%**	**5.2%**	**8.4%**	**12.8%**	**18.5%**	**27.2%**	**38.4%**
**Vascular Disease**										
Abdominal Aortic Aneurysm	Never	<	<	<	<	<	<	<	<	<
	Current	<	<	<	<	<	<	<	0.1%	0.1%
Coronary Heart Disease	Never	<	0.1%	0.1%	0.2%	0.3%	0.5%	0.9%	1.5%	2.7%
	Current	<	0.1%	0.3%	0.5%	1.0%	1.6%	2.1%	2.9%	4.3%
Heart Failure	Never	<	<	<	<	<	0.1%	0.1%	0.2%	0.4%
	Current	<	<	<	<	0.1%	0.1%	0.2%	0.4%	0.7%
High Blood Pressure [a]	Never	<	<	<	0.1%	0.1%	0.1%	0.2%	0.3%	0.5%
	Current	<	<	0.1%	0.1%	0.2%	0.2%	0.3%	0.5%	0.8%
Stroke	Never	<	<	0.1%	0.1%	0.1%	0.2%	0.3%	0.6%	1.2%
	Current	<	<	0.1%	0.1%	0.2%	0.3%	0.5%	0.9%	1.5%
**Cancer**										
Breast	Never	<	0.1%	0.1%	0.2%	0.3%	0.4%	0.4%	0.6%	0.7%
	Current	<	0.1%	0.2%	0.2%	0.3%	0.4%	0.5%	0.7%	0.8%
Cervical	Never	<	<	<	<	<	<	<	<	<
	Current	<	<	<	<	0.1%	<	0.1%	0.1%	<
Colon and Rectum	Never	<	<	0.1%	0.1%	0.1%	0.2%	0.2%	0.3%	0.4%
	Current	<	<	0.1%	0.1%	0.2%	0.2%	0.3%	0.4%	0.5%
Lung and Bronchus	Never	<	<	<	0.1%	0.1%	0.2%	0.2%	0.3%	0.5%
	Current	<	0.1%	0.2%	0.5%	1.2%	2.2%	3.4%	5.1%	6.8%
Ovarian	Never	<	<	<	0.1%	0.1%	0.1%	0.2%	0.3%	0.3%
	Current	<	<	<	0.1%	0.1%	0.1%	0.2%	0.2%	0.3%
Pancreas	Never	<	<	<	<	0.1%	0.1%	0.2%	0.3%	0.4%
	Current	<	<	<	0.1%	0.2%	0.3%	0.4%	0.6%	0.8%
**Lung Disease**										
COPD	Never	<	<	<	<	0.1%	0.1%	0.2%	0.3%	0.6%
	Current	<	<	0.1%	0.3%	0.7%	1.4%	2.4%	4.4%	7.3%
**Infection**										
AIDS	Never	<	<	<	<	<	<	<	<	<
	Current	<	<	<	<	<	<	<	<	<
Pneumonia/Flu [a]	Never	<	<	<	<	0.1%	0.1%	0.1%	0.2%	0.4%
	Current	<	<	<	0.1%	0.1%	0.2%	0.2%	0.4%	0.6%
**Accidents and Injury**										
Accidents	Never	0.3%	0.3%	0.3%	0.4%	0.4%	0.3%	0.3%	0.3%	0.5%
	Current	0.3%	0.3%	0.3%	0.4%	0.4%	0.3%	0.3%	0.3%	0.4%
Homicide	Never	<	<	<	<	<	<	<	<	<
	Current	<	<	<	<	<	<	<	<	<
Suicide	Never	0.1%	0.1%	0.1%	0.1%	0.1%	0.1%	0.1%	0.1%	0.1%
	Current	0.1%	0.1%	0.1%	0.1%	0.1%	0.1%	0.1%	0.1%	<
**Diabetes [a]**	**Never**	<	<	**0.1%**	**0.1%**	**0.1%**	**0.2%**	**0.3%**	**0.5%**	**0.6%**
	**Current**	<	<	**0.1%**	**0.1%**	**0.2%**	**0.2%**	**0.4%**	**0.5%**	**0.7%**
**Neurological Disease**										
Alzheimers Disease	Never	<	<	<	<	<	<	0.1%	0.3%	0.9%
	Current	<	<	<	<	<	<	0.1%	0.3%	0.8%
Parkinsons Disease	Never	<	<	<	<	<	<	<	0.1%	0.3%
	Current	<	<	<	<	<	<	<	<	0.1%

© 2023 Woloshin S et al. *JAMA Network Open*.

Tailored charts can be created on the Know Your Chances website (https://www.knowyourchances.cancer.gov)

Sources: National Center for Health Statistics (NCHS, using years 2016-2018) (mortality); National Health Interview Survey (NHIS, smoking prevalence), and the NHIS linked mortality file (NHIS-LMF); and multiple cohort studies (Carter, NEJM, 2015; CPS II Nutrition cohort, NIH-AARP cohort, and Nationwide American Cancer Society Prospective Cohorts), (relative risks). For methodologic details see [CITATION FOR ARTICLE UNDER REVIEW]
Risk estimates are based on the year range 2016-2018.

* Numbers in columns do not add up to "all causes" because there are many other causes of death besides those listed.

< Risk is less than 0.1%.

[a] Rules used by the National Center for Disease Statistics (NCHS) may result in over- and under-counting of some underlying causes of death. Diabetes and high blood pressure deaths, for example, are probably under-counted: they are often reported as contributing factors rather than as the underlying cause of death because of uncertainty in the chain of events leading to death. Flu deaths are probably over-counted since many pneumonia deaths, completely unrelated to the flu, are nevertheless attributed to it. Because the conditions are difficult to disentangle, we present a combined flu/pneumonia category.

© 2023 Woloshin S et al. *JAMA Network Open*.

eFigure 1. The Effect of Smoking Status on the Order of the Top 5 Causes of Death and Absolute Risk of Death Over 10 Years for a 60-Year-Old White Man

The yellow shading shows the effect on a smoking related cause (lung cancer), and the blue shading shows the effect on a non-smoking related cause (accidents). Tailored charts can be created on the Know Your Chances website (https://www.knowyourchances.cancer.gov). For methodologic details see the main article.

White Male, Age 60 until Age 70

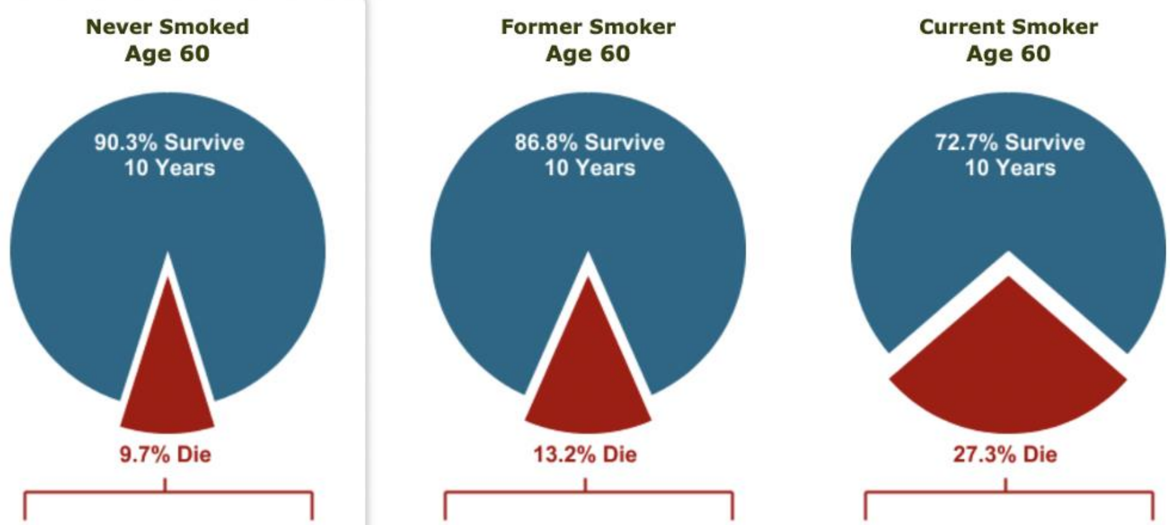

Never Smoked, Age 60

Your chance of dying from various causes of death **in the next 10 years** starting at **Age 60** are:

	**Total**	**9.7%**
1.	Coronary Heart Disease	2.0%
2.	Accidents	0.7%
3.	Diabetes	0.5%
4.	Stroke	0.5%
5.	High Blood Pressure	0.4%
6.	Colon and Rectum Cancer	0.3%
7.	Pancreas Cancer	0.3%
8.	Lung and Bronchus Cancer	0.3%
9.	Suicide	0.3%
10.	Chronic Liver Disease and Cirrhosis	0.3%
	Remaining Causes	4.2%

Former Smoker, Age 60

Your chance of dying from various causes of death **in the next 10 years** starting at **Age 60** are:

	**Total**	**13.2%**
1.	Coronary Heart Disease	2.7%
2.	Lung and Bronchus Cancer	0.9%
3.	COPD	0.8%
4.	Accidents	0.7%
5.	Diabetes	0.6%
6.	Stroke	0.5%
7.	Colon and Rectum Cancer	0.4%
8.	Chronic Liver Disease and Cirrhosis	0.4%
9.	High Blood Pressure	0.4%
10.	Liver Cancer	0.4%
	Remaining Causes	5.5%

Current Smoker, Age 60

Your chance of dying from various causes of death **in the next 10 years** starting at **Age 60** are:

	**Total**	**27.3%**
1.	Coronary Heart Disease	5.0%
2.	Lung and Bronchus Cancer	3.8%
3.	COPD	2.3%
4.	Chronic Liver Disease and Cirrhosis	1.0%
5.	Diabetes	0.7%
6.	Accidents	0.6%
7.	High Blood Pressure	0.6%
8.	Stroke	0.5%
9.	Liver Cancer	0.5%
10.	Pancreas Cancer	0.5%
	Remaining Causes	11.9%

© 2023 Woloshin S et al. *JAMA Network Open*.

Source: US Mortality Files, National Center for Health Statistics, Centers for Disease Control and Prevention.
Risk estimates are based on the year range 2016-2018.

Be cautious interpreting exact rankings for less common causes of death. Because of the wider "margin of error", closely-ranked causes might be tied or even flipped.

The cancer sites in the tables are ranked based on very precise estimates of the risk of death (ie, 4 decimal place precision). You can view results at this level of precision by selecting the 'Show maximum precision ...' option.

⁻ Risk is less than <0.0001 and cannot be ranked.

© 2023 Woloshin S et al. *JAMA Network Open*.

eFigure 2. Overview of Computational Details for Partitioning Mortality Rates by Smoking Status for Smoking-Related and Non–Smoking-Related Causes of Death

Overall age-specific mortality rate of death can be written as the weighted average of the age-specific rates for current, former and never smokers, where the weights reflect the prevalence of the population with each smoking status.

(A) Smoking-related causes of death

Derivation of M(D; age; current), M(D; age; former), M(D; age; never) [a]

Equation 1
Using law of total probability:
M(D; age) = M(D; age; current)·p(age; current) + M(D; age; former)·p(age; former) + M(D; age; never)·p(age; never)

Equation 2[b]
M(D; age; current) = M(D; age; never)·RR(D; age; current)

Equation 3
M(D; age; former) = M(D; age; never)·RR(D; age; former)

Equation 4[c]
Substituting equations 2 and 3 into equation 1, and rearranging terms indicates:
M(D; age; never) = M(D; age) / {p(age; current)·RR(D; age; current) + p(age; former)·RR(D; age; former) + p(age; never)}

Derivation of M(O; age; current), M(O; age; former), M(O; age; never) [d]

For the 10 smoking-related causes, whose RRs were derived from the NHIS-LMF, the rates M(O; age; current), M(O; age; former) and M(O; age; never) were obtained using Equations 1-4 by substituting O for D. For the other 22 smoking-related causes we did not have the RRs for the complements and so to derive M(O; age; current), M(O; age; former) and M(O; age; never) for these causes we first estimated the partitioned all-cause age-specific mortality rates, M(all-cause; age; current), M(all-cause; age; former) and M(all-cause; age; never), by substituting all-cause mortality for D in Equations 1-4. Then, we derived M(O; age; current) = M(all-cause; age; current) – M(D; age; current) with analogous equations for M(O; age; former) and M(O; age; never).

[a] D is a smoking-related cause of death

[b] M(D; age) is the *overall* (all three smoking status groups combined) age-specific mortality rate for cause D and a given age interval; p(age; current), p(age; former) and p(age; never) represent the age-specific prevalences of smoking status; M(D; age; current), M(D; age; former); M(D; age; never) are age-specific mortality rates for a given cause D and a given age interval for current, former and never smokers, respectively

[c] RR(D; age; current) and RR(D; age; former) are the relative risks (RRs) of dying from cause D for current and former smokers relative to never smokers (with RR(D; age; never) = 1 by definition)

[d] O is a complement of a smoking-related cause of death (i.e., all causes except D)

© 2023 Woloshin S et al. *JAMA Network Open*.

(B) Non-smoking-related causes of death

Derivation of M(D*; age; current), M(D*; age; former), M(D*; age; never) [a]

Equation 1[b]
Because D* is not smoking-related,
M(D*; age; current) = M(D*; age; former) = M(D*; age; never) = M(D*; age)

Derivation of M(O*; age; current), M(O*; age; former), M(O*; age; never) [c]

Equation 2[d]
Using equation 1:
M(O*; age; current) = M(all-cause; age; current) – M(D*; age; current) =
M(all-cause; age; current) – M(D*; age).

M(O*; age; former) and M(O*; age; never) are derived using analogous equations.

[a] D* is a non-smoking-related cause of death

[b] M(D*; age) is the *overall* (all three smoking status groups combined) age-specific mortality rate for a given non-smoking-related cause of death D* and a given age interval; M(D*; age; current), M(D*; age; former); M(D*; age; never) are age-specific mortality rates for a given cause D* and a given age interval for current, former and never smokers, respectively

[c] O* is any cause except D*

[d] M(O*; age; current), M(O*, age; former), M(O*, age, never) are the age-specific mortality rates for a cause O* and a given age interval for current, former and never smokers; M(all-cause; age; current), M(all-cause; age; former); M(all-cause; age; never) are age-specific mortality rates for all-cause mortality and a given age interval for current, former and never smokers, respectively

Note: Former smoker: Smoked >100 cigarettes in their lifetime and quit >2 years before the NHIS interview

© 2023 Woloshin S et al. *JAMA Network Open*.

eReferences

1. DevCan - Probability of Developing or Dying of Cancer. National Cancer Institute. Division of Cancer Control & Population Sciences. Accessed 2.27.2023. https://surveillance.cancer.gov/devcan/.
2. Fay M, Pfeiffer R, Cronin K, Le C, Feuer E. Age-conditional probabilities of developing cancer. . *Stat Med* 2003;22:1837-1848.
3. Fay, M.P. Estimating age conditional probability of developing disease from surveillance data. Popul Health Metrics 2, 6 (2004). https://doi.org/10.1186/1478-7954-2-6. *Popul Health Metrics*
4. Fine, J. P. and Gray, R. J. (1999). A proportional Hazards Model for Subdistribution of a Competing Risk. Journalof the American Statistical Association 94, 496–509.
5. Lau B, Cole SR, Gange SJ. Competing risk regression models for epidemiologic data. Am J Epidemiol. 2009 Jul 15;170(2):244-56. doi: 10.1093/aje/kwp107. Epub 2009 Jun 3. PMID: 19494242; PMCID: PMC2732996.
6. Carter B, Abnet C, Feskanich D, et al. Smoking and Mortality — Beyond Established Causes. *N Engl J Med.* 2015;372:631-640.
7. National Cancer Institute. Division of Cancer Epidemiology & Genetics. NIH-AARP Diet and Health Study. Accessed June 7, 2021 . https://dietandhealth.cancer.gov/.
8. American Cancer Society. Cancer Prevention Study II (CPS II). Accessed June 7, 2021. https://www.cancer.org/research/population-science/cancer-prevention-and-survivorship-research-team/acs-cancer-prevention-studies/cancer-prevention-study-2.html.
9. Thun M, Apicella L, Henley S. Smoking vs Other Risk Factors as the Cause of Smoking-Attributable Deaths: Confounding in the Courtroom. *JAMA.* 2000;284:706-712.
10. Korn EL, Graubard BI, Midthune D. Time-to-event analysis of longitudinal follow-up of a survey: choice of the time-scale. Am J Epidemiol. 1997 Jan 1;145(1):72-80. doi: 10.1093/oxfordjournals.aje.a009034. PMID: 8982025.

© 2023 Woloshin S et al. *JAMA Network Open*.
