# Supplementary material for: Updating the Know Your Chances Website to Include Smoking Status as a Risk Factor for Mortality Estimates
Source: JAMA Netw Open. 2023 Jun 8;6(6):e2317351. doi: 10.1001/jamanetworkopen.2023.17351 (PMC10251216; doi:10.1001/jamanetworkopen.2023.17351)
Supplement: Supplement 2. — Data Sharing Statement [file jamanetwopen-e2317351-s002.pdf]

Data Sharing Statement

Woloshin. Updating the Know Your Chances Website to Include Smoking Status as a Risk Factor for Mortality Estimates. *JAMA Netw Open*. Published June 08, 2023. doi:10.1001/jamanetworkopen.2023.17351

Data
Data available: No
